# Vacancy-induced modifications to the electronic structure of barium lanthanum cobaltite: density functional theory calculations

**DOI:** 10.1039/d6ra05408k

**Published:** 2026-09-14

**Authors:** Francis Oseko, Aleksandra Mielewczyk-Gryń, Szymon Winczewski

**Affiliations:** a Institute of Nanotechnology and Materials Engineering, Gdańsk University of Technology Gabriela Narutowicza 11/12 80-233 Gdańsk Poland francis.oseko@pg.edu.pl

## Abstract

This work investigates the influence of oxygen and cobalt vacancies on the structural, electronic and magnetic properties of cubic barium lanthanum cobaltite (BLCO). A novel multi-objective evolutionary algorithm is applied in the generation of dedicated pseudopotentials which are later used in the density functional theory (DFT) calculations performed for the perfect and defective BLCO structures. The Hubbard + *U* correction is applied to La, Co and O atoms and the accuracy of the developed methodology is assessed through the cohesive energy and equation of state calculations, performed for the perfect BLCO. The results are found to be in agreement with the experimental observations and earlier all electron calculations. The validated methodology is applied in the investigation on defective BLCO, considering three types of defects: a single oxygen vacancy, transverse-oxygen (*trans*-O) vacancy pair, and complex oxygen–cobalt–oxygen vacancy, all in charged and neutral states. The obtained results reveal that all studied defects considerably affect the structural, electronic and magnetic properties of BLCO, which we comprehensively characterize. Among others, we show that in BLCO the redistribution of charge has a mixed character, with the introduction of defects resulting in both charge localization and delocalization. We attribute this to competing mechanisms originating from the half-metallicity of BLCO, its ferromagnetic ordering, and the tendency of Co atoms to undergo reduction. Our DFT + *U* calculations show only a mild reduction of the Co atoms near the defect, and this observation is contrary to the strong reduction predicted by defect chemistry models.

## Abstract 2

This work investigates the influence of oxygen and cobalt vacancies on the structural, electronic and magnetic properties of cubic barium lanthanum cobaltite (BLCO).

## Graphical abstract

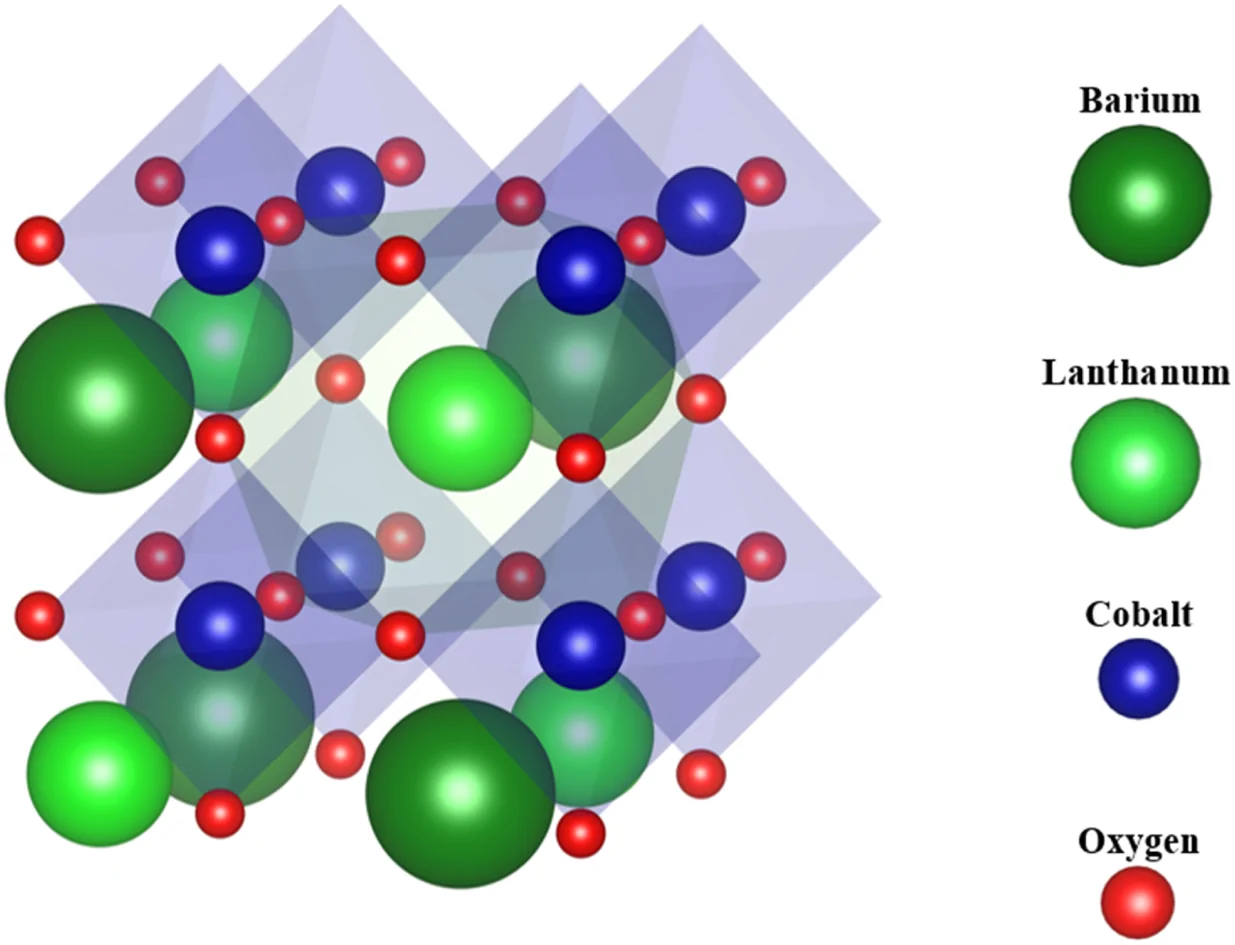

## 1 Introduction

In recent years, extensive experimental studies have been carried out on the structure, electrical and magnetic properties of the Ba_0.5_La_0.5_CoO_3−*δ*_ (BLCO) perovskite. According to Szpunar *et al.*, a solid solution of BaCoO_3_ and LaCoO_3_ makes up the BLCO structure.^1^ BaCoO_3_ has a hexagonal structure, possessing chains of Co ions which are set apart by Ba ions. The triangle enclosures of oxygen ions are orthogonal to the Co chains where two CoO_6_ octahedra share an individual triangle.^1–3^ On the other hand, LaCoO_3_ has a rhombohedral structure where the placement of oxygen ions around the [111] axis alters the crystal symmetry from cubic to rhombohedral.

**Fig. 1:**
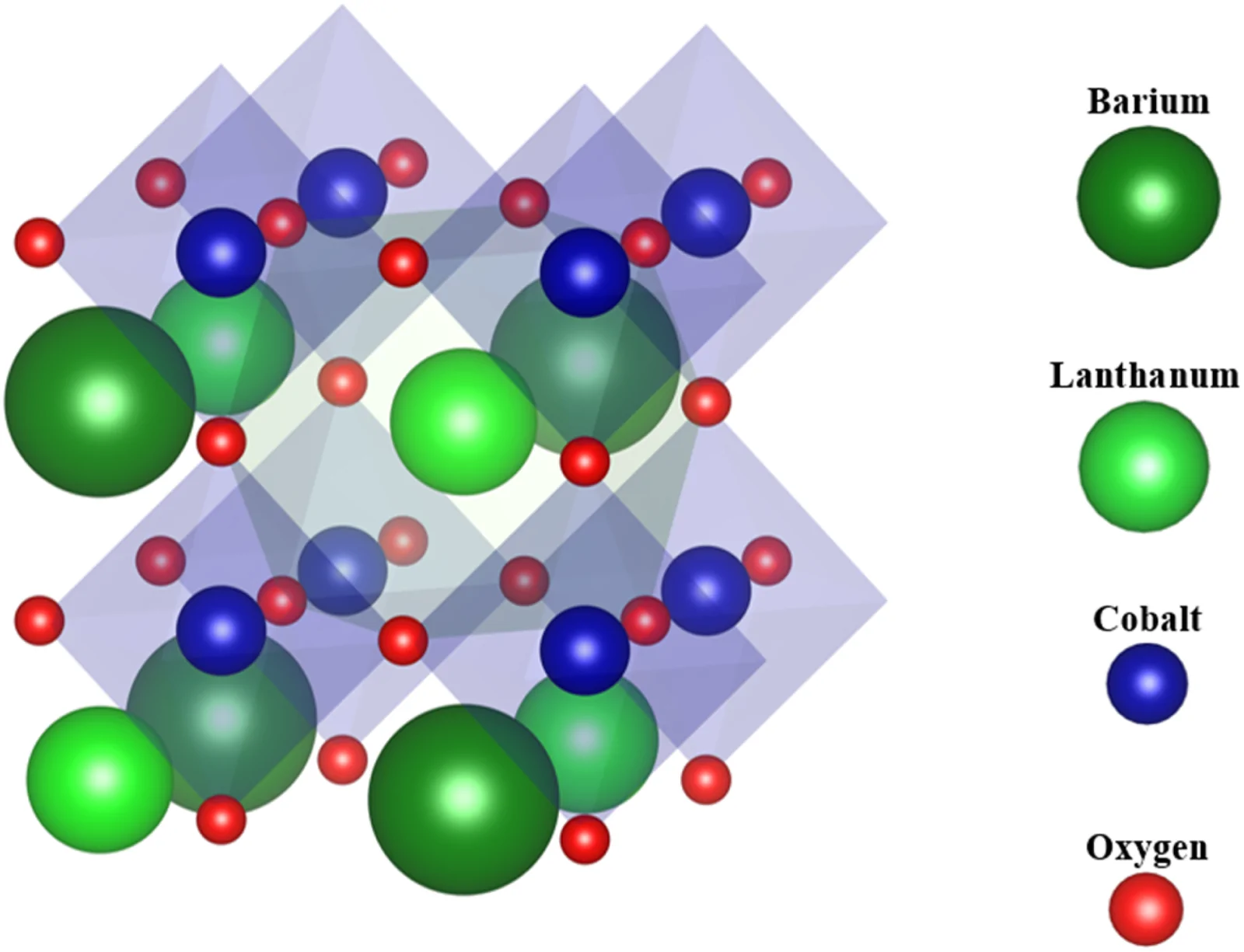
Perfect BLCO supercell used for all DFT calculations with the Ba and La atoms arranged in an alternating pattern.

An increase in Ba concentration in LaCoO_3_ induces a phase transformation from R-3c to the cubic Pm3̲m crystal structure. Therefore, at *x* = 0.5 the BLCO structure is cubic as shown in Fig. 1 and for *x* ≥ 0.7 it becomes a mixture of hexagonal and cubic structures.^1,3^ To improve the catalytic properties of BLCO, several approaches have been adopted in the past, such as the exsolution of nanoparticles, and chemical and physical vapor deposition of nanoparticles.^4,5^ Both chemical and physical vapor deposition methods are complex and result in weak nanoparticle–perovskite interaction, irreversible nanoparticle degradation and non-uniform distribution of nanoparticles.^4^

The exsolution of nanoparticles, on the other hand, allows for simple, controllable and uniform distribution of nanoparticles on both A-site and B-site terminated planes. The nanoparticles obtained *via* exsolution are also strongly socketed on the perovskite with the potential for regeneration upon degradation.^4,6^ Exsolution of nanoparticles entails the removal of A-site or B-site cations from the lattice by reduction or oxidation method.^6^ The exsolved cations pair with oxygen vacancies and migrate to the surface, where they nucleate socketed metallic or oxide nanoparticles. The exsolution of cobalt oxide nanoparticles on the surface of BLCO has been shown to be effective in increasing the catalytic activity, *e.g.*, the oxygen reduction reaction in fuel cell cathodes.^4,5,7^ However, the exsolution process of nanoparticles changes the structural, electronic and magnetic properties of BLCO.^4,6^ The underlying reason is that the exsolution process introduces various point defects into the lattice of the host material.

Among these defects, oxygen vacancies play a particularly important role.^4,6^ Since they facilitate the migration of Co atoms toward the surface, their presence is required for the exsolution process to proceed. This transport often occurs as the migration of a complex defect, formed by a Co atom surrounded by two oxygen vacancies.^4–6^ This mechanism provides a source of Co atoms for the exsolved nanoparticles. Because it effectively removes the Co atoms from the lattice, it naturally leads to the formation of Co vacancies. They typically appear as complex defects, co-joining with two oxygen vacancies.^4,5^

Recent reports have shown that the exsolution-related defects specified above strongly influence the properties of BLCO.^4,5^ For example, the experimental studies on Lu doped BLCO and Fe doped BLCO have shown that the exsolution of nanoparticles result in lattice distortions and the formation of secondary phases.^5,8^ In extreme situations of oxygen loss in cubic BLCO, as happens during exsolution, oxygen vacancy ordering and lattice restructuring can occur in the host perovskite to form metastable cobaltite-based perovskites or phases of layered tetragonal BLCO.^1^ Moreover, the removal of B-site cobalt cations and an increase in electron concentration reduces the concentration of electron–holes within the lattice which alters the electronic conductivity.^1,4^ During the exsolution process, the reduction of low-spin Co^3+^ to high-spin Co^2+^ is expected to increase the number of unpaired electrons and enhance double exchange interactions which could promote ferromagnetic ordering in BLCO.^1,9,10^

To the best of our knowledge, there is only one computational study which focused on the BLCO. It was conducted by Maria *et al.*,^11^ who investigated the electronic structure and magnetic properties of the perfect BLCO, using the all-electron FP-LAPW + lo method. Building on the above observations, in this work we supplement the existing knowledge by studying the structural, electronic and magnetic properties of the defective BLCO.

In particular, the scope of this study first entailed the development of the methodology and its validation. In this step, we generated from scratch dedicated pseudopotentials, which were tailored for an accurate representation of BLCO. In the generation of norm-conserving pseudopotentials we employed our novel approach, which uses multi-objective evolutionary algorithm.^12,13^ In the next step, we verified the developed methodology by performing standard DFT and DFT + *U* calculations for the perfect BLCO, and comparing their predictions against the experiment. In the final step we investigated the influence of vacancies. We considered three types of defects which were single oxygen vacancy, *trans*-O vacancy pair (two oxygen vacancies), and a complex defect composed of oxygen–cobalt–oxygen vacancy. We performed geometry relaxations for these defects, and later studied their influence on the structural, electronic and magnetic properties of BLCO.

## 2 Theoretical background

In this section we discuss from a theoretical perspective defects in BLCO. First, we focus on the perfect BLCO, explaining how Co^3+^ and Co^4+^ ions are created within its structure. We also briefly discuss the crystal field splitting and exchange splitting of the CoO_6_ octahedron. The above phenomena are important for understanding the electronic structure of the perfect BLCO. Having this basis is fundamental for describing the influence of defects, which we discuss from a theoretical perspective later on in this section. We focus only on the exsolution-related defects namely; single oxygen vacancy, *trans*-O vacancy pair (two oxygen vacancies on opposite sides of a cobalt atom), and complex oxygen–cobalt–oxygen defect. The description that we present here are the theoretical predictions of defect chemistry.^14^ Later on in this work, these defect chemistry predictions will be confronted against theoretical predictions made based on DFT calculations.

### 2.1 Electronic structure of BLCO

It is known^1,3^ that in BLCO, cobalt at the B-site typically exists in two oxidation states: +3 and +4. The existence of these oxidation states can be explained by considering the formation of BLCO from LCO (lanthanum cobaltite, with the formula LaCoO_3_), as a result of barium substitution on the lanthanum site.

In LCO, a rhombohedral perovskite at room temperature,^10,15,16^ cobalt exists in the +3 oxidation state. The aliovalent substitution of La^3+^ with Ba^2+^ (
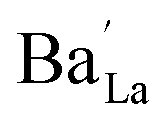
 in Kroger–Vink notation) in LCO results in the formation of electron holes and subsequent creation of Co^4+^ (
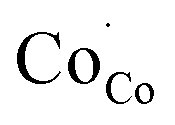
) which ensures charge neutrality. This reaction can be written with the following equation (also in Kroger–Vink notation):^14^1
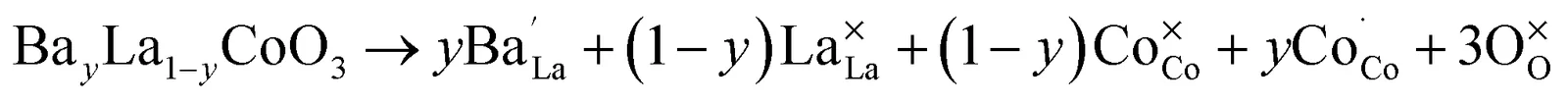
where Co^×^_Co_, La^×^_La_ and O^×^_O_ represent neutral sites within the lattice, and *y* = 0.5 represents the fraction of substituted La cations.

As a result, the A-site of the BLCO is occupied with La^3+^ and Ba^2+^ cations, while the B-site contains a mixture of Co^3+^ and Co^4+^ cations. As seen in Fig. 1, the O^2−^ anions form a network of octahedra surrounding the B-site ions (Co cations). Here, we underline the presence of multivalent Co cations in BLCO, a feature which needs to be accounted for in a physically sound theoretical model.^1,2^

The description of crystal field and exchange splitting here plays a crucial role in understanding many of BLCO's properties, especially those related to its electronic structure. The underlying reason is that in BLCO the valence and conduction bands are mostly formed from O 2p and Co 3d states, with the latter undergoing two previously mentioned splitting effects.

**Fig. 2:**
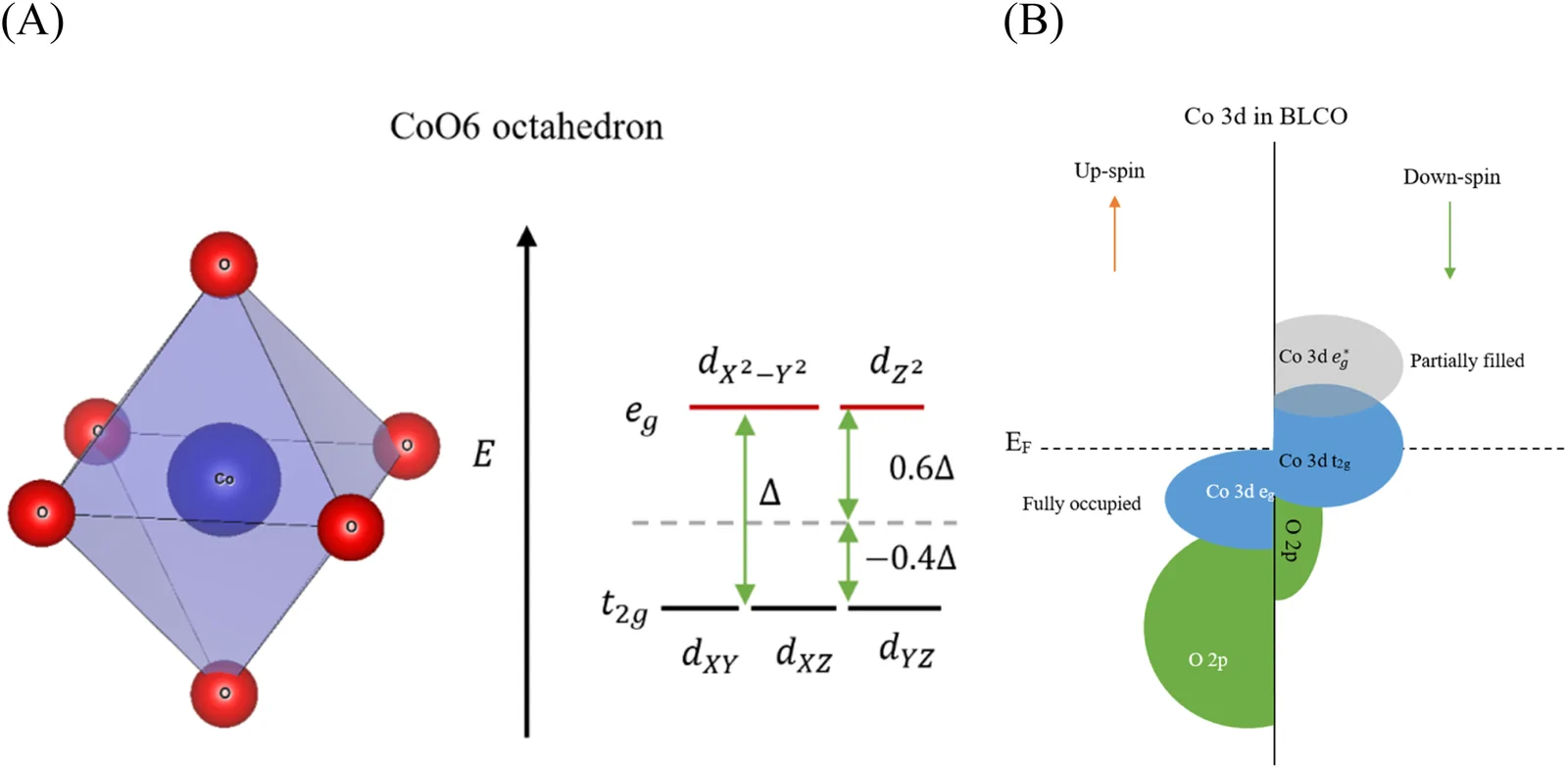
(A) Schematic illustrations of the cobalt octahedron complex. (B) Influence of the crystal field and exchange splitting on the Co 3d levels.^17^

In the first idealized situation (Fig. 2A), the crystal field splitting eliminates the degeneracy of the Co 3d states, shifting three of them (d_*XY*_, d_*XZ*_ and d_*YZ*_) to lower energy, and the remaining two (d_*X*^2^−*Y*^2^_ and d_Z^2^_) to higher energy. This effect is a consequence of the electrostatic interactions between the Co 3d orbitals and O ligands arranged in an octahedral symmetry. As a result, two groups of levels are produced, e_g_ and t_2g_, as shown in Fig. 2A. Here, g means the presence of symmetry with respect to the octahedron center. The magnitude of this effect is quantified with the delta parameter (Δ), which is the energy separating e_g_ and t_2g_ levels. The crystal field splitting occurs such that the overall energy remains unchanged.^15,18^

Exchange splitting, on the contrary, refers to an intra-atomic phenomenon which is due to electron–electron exchange interaction. It minimizes the energy of the CoO_6_ octahedron by separating in the energy domain the electrons with different spin alignments (up and down).^6,15,18^

In a real compound, like BLCO, the occurrence of crystal field and exchange splitting together produce a picture which is more sophisticated than that shown in Fig. 2A. For example, both splitting effects may lead to a situation where e_g_ and t_2g_ levels are overlapping in the energy domain, as shown in Fig. 2B.

### 2.2 Exsolution-related defects in BLCO

According to defect chemistry,^14^ the formation of a single oxygen vacancy may result in either charge localization and delocalization. The underlying reason is that the two electrons, which remain in the crystal after the removal of the oxygen atom may either populate a localized level, producing a localized defect state, or move to the conduction band of the host material. These situations can be described with the following reaction equations, written in the Kroger–Vink notation:2
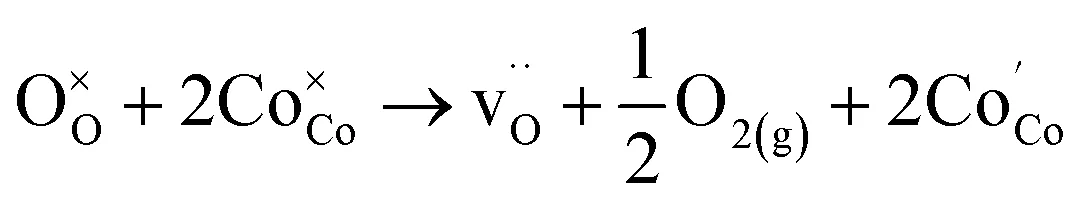
and3
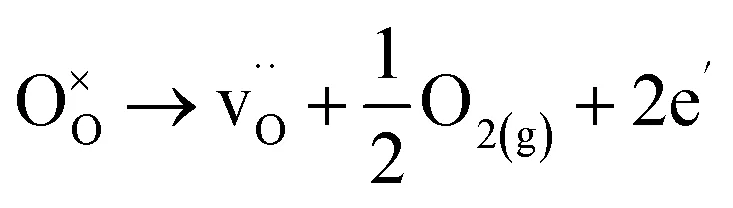
In the above equations, 
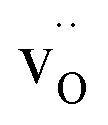
 represents a positively charged oxygen vacancy, e′ is a released electron, and 
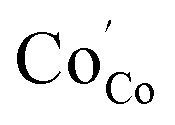
 is a reduced cobalt on its lattice site. The charge localization causes the chemical reduction of two cobalt ions from Co^4+^ to Co^3+^, as seen in eqn (2). Eqn (3) describes the case of electron delocalization in the conduction band.^14^ Notably, both reactions can occur at the same time, with one electron reducing Co, and one moving to the conduction band.

Moreover, the clustering of oxygen vacancies around the Co cation is an important mechanism of the exsolution process.^5,6^ It may occur through the formation of two oxygen vacancies on the opposite vertices of a CoO_6_ octahedron. This process leads to a creation of the so-called trans O-vacancy pair^19^ and from the perspective of defect chemistry, it may also result in either charge localization and delocalization. The first situation can be described with the following reaction equation:4
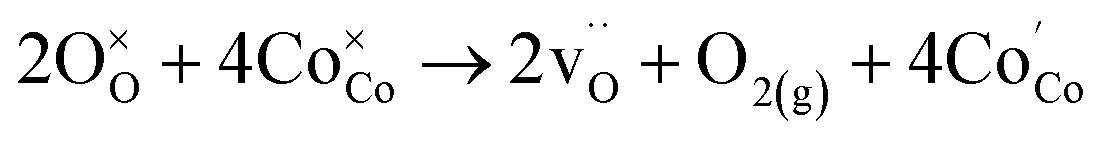
where four electrons donated by two removed oxygen atoms reduce four Co^4+^ ions to Co^3+^. The charge delocalization situation is described with the equation:5
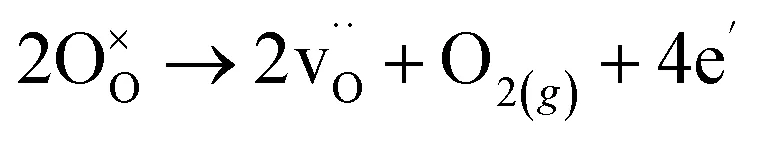
which is an analogue of eqn (3), as it also describes the process of populating the conduction band.^14^

The last defect involved in the exsolution process is a complex oxygen–cobalt–oxygen vacancy. It is formed when the central cobalt atom is removed in the trans O-vacancy pair. The formation of these complex defects is a source of atoms for the exsolved CoO oxide nanoparticles.^4,5^ The related reaction can be described with the following equation:6
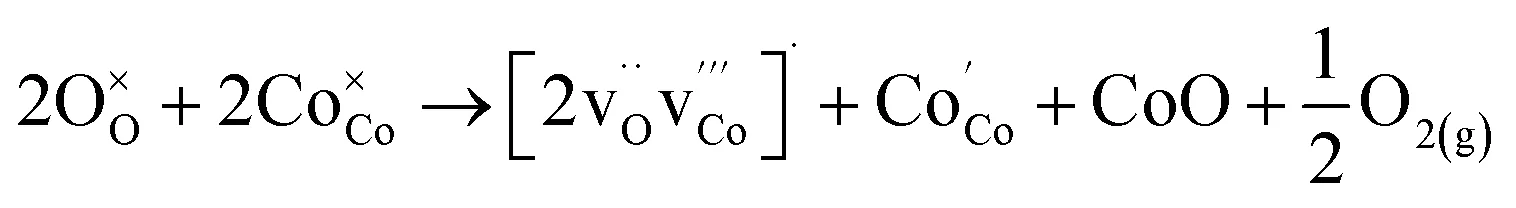
In this reaction two oxygen atoms and one cobalt atom are removed from the material, producing the mentioned complex defect 
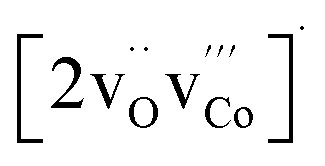
, which effectively carries a 1+ charge.^14^
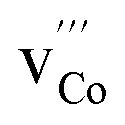
 represents a vacant cobalt site. The reaction involves the reduction of two Co sites, where one is reduced from +3 to 0, and another is reduced from +4 to +3. Effectively, this reaction serves as a source of the neutral Co (and O) atoms, which later form CoO nanoparticles.^4–6^

There is also a possibility that the above reaction will proceed in two slightly different scenarios. The first one is described with the following reaction equation:7
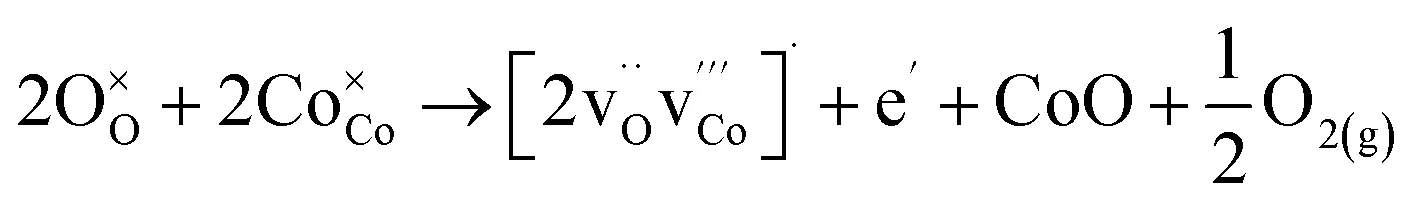
In this variant, the same complex defect 
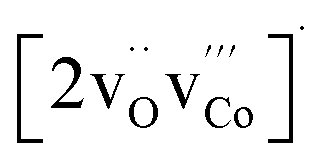
 is formed, which also carries the 1+ effective charge. However, there is no Co^4+^ to Co^3+^ reduction. Instead, the extra electron remaining after the reaction populates the conduction band.^14,20^

In the second alternative scenario, which can be summarized with the following reaction equation:8
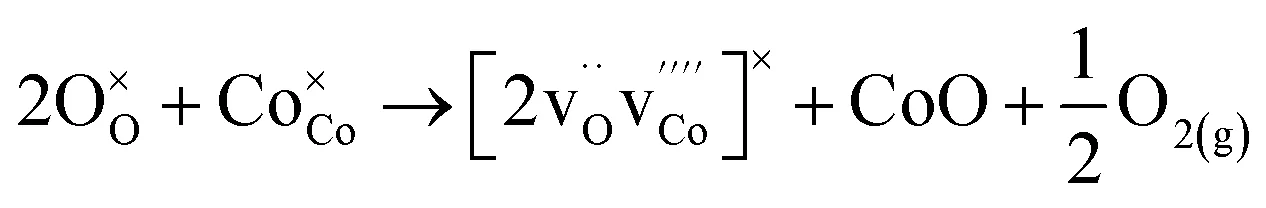
The complex oxygen–cobalt–oxygen vacancy 
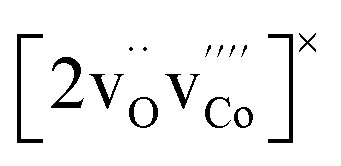
 is also formed, however, it has zero effective charge. In this case, the first Co^4+^ site is reduced to Co^0^ while the second Co site is not involved in the reaction. In principle, in the above three reactions, the Co^3+^ to Co^2+^ reduction may also occur. However, it leads to the formation of unstable CoO_6_ octahedra^4,14,20^ and therefore, the related reactions are not preferable.

## 3 Methodology

### 3.1 Generation of dedicated pseudopotentials

BLCO contains elements which are difficult to describe at the DFT level. To improve our theoretical description at the very beginning of our study we generated new pseudopotentials for Ba, La, and Co atoms, and also for O. The aim was to obtain pseudopotentials that would better represent the cores of these atoms in the studied compound, than the pseudopotentials available in the standard pseudopotential libraries. These typically provide general purpose pseudopotentials, designed to allow a sufficiently accurate description of atoms in various chemical environments, including compounds with different bonding types. This universality, however, comes at the cost of accuracy when representing specific compounds, and much better representations can be obtained with tailored pseudopotentials.

In the generation of dedicated pseudopotentials we used our novel approach. Within it, the finding of a good pseudopotential is formulated as a multi-objective optimization problem. The variables which are optimized are all the pseudopotential's design-parameters, like pseudization radii, numbers and truncations of the basis functions used in the pseudopotential's representation, *etc.* The objectives which guide the optimization are various measures characterizing the pseudopotential, its hardness, accuracy, and transferability. These objectives are calculated by checking how the pseudopotential performs in the description of various electronic configurations of an isolated atom. In our approach, the generation of a dedicated pseudopotential is achieved by a proper choice of the tested electronic configurations. This set contains configurations which are met in the studied compound, like highly ionic Ba^2+^, La^3+^, Co^3+/4+^ and O^2−^ configurations found in the BLCO.

In our approach the resulting multi-objective optimization problem is addressed with the MOEA/D-DE algorithm.^12,13^ It allows efficient searching of the optimization space and produces high quality Pareto fronts, containing various Pareto optimal solutions (pseudopotentials in our case), corresponding to various compromises between the considered objectives. In the supplementary information we provide the pseudopotentials used in the form of the RECPOT files and OPIUM param files.^21^

We note that the pseudopotentials which we generated and used in this work are scalar relativistic norm-conserving pseudopotentials. They are of the Rappe–Rabe–Kaxiras–Joannopoulos designed optimized type^22–24^ and are in the Kleinman–Bylander fully non-local separable form.^25^ For improved accuracy and transferability, all pseudopotentials include partial core charge for non-linear core corrections.^26,27^ In addition, the pseudopotentials for La, Ba and Co include semi-core states in the valence.

### 3.2 Computational framework

All the quantum mechanical calculations reported in this work were done by employing plane wave density functional theory (PW DFT) using Cambridge Serial Total Energy Package (CASTEP).^28^ The generalized gradient approximation (GGA) was adopted with the Perdew–Burke–Ernzerhof (PBE) exchange–correlation functional.^29^

All the calculations used a supercell consisting of 2 × 2 × 2 repetitions of a 5-atom cubic cell (see Fig. 1). It had 40 atoms in total with an alternating arrangement of A-site ions (Ba and La). Periodic boundary conditions were applied along all Cartesian directions. The experimental lattice constant of 3.8845 Å was set for a single unit cell.

The plane wave cutoff energy was set to 1600 eV. This choice is motivated by the use of dedicated norm-conserving pseudopotentials, which are accurate but computationally costly because of their high hardness. In the self-consistent field procedure, the stopping criterion (tolerance for accepting convergence of the total energy) was set to 10^−6^ eV, which ensured well-converged total energies and forces.

In the EOS calculations done for perfect BLCO and geometry relaxations performed for defective systems, we used a 4 × 4 × 4 Monkhorst–Pack *k*-point grid. However, in calculations of density of states and band structures we used finer grids, reaching 21 × 21 × 21.

In the norm-conserving pseudopotentials that we used, the semi-core states are included in the valence and treated explicitly. In the case of individual elements, the following number of valence electrons were represented: 10 for Ba, 21 for La, 17 for Co, and 6 for O. This results in an explicit representation of 364 electrons in the 2 × 2 × 2 supercell adopted. In addition, the calculations were carried out in a spin polarized scheme with each spin channel treated separately. Employing this scheme is essential for correctly representing both the ferromagnetic ordering and half-metallicity^11^ of BLCO, as well as the changes in its magnetic properties induced by defects. To obtain the correct magnetic properties the initial spins of the individual atoms were adjusted and later optimized in the SCF procedure, a feature implemented in CASTEP.^28^

Standard DFT fails to correctly describe the band structure of many compounds, especially oxides and materials with strongly-correlated electrons. Therefore, in this work, we overcome the above shortcoming by using a corrective approach, namely DFT + *U*. In DFT + *U*, the Hubbard model is incorporated to improve the theoretical description of strongly correlated electronic states (*e.g.*, 4f orbitals of La and 3d orbitals of Co), providing more accurate ground state and total energies.^3,28,29^

The application of the DFT + *U* method requires choosing the *U* parameters. In our choice we followed the work by Maria *et al.*^11^ as a guideline. This led us to the following choice of the *U* parameters: 5 eV for Co 3d, 5 eV for La 5d, and 8 eV for O 2p. This set of parameters resulted in consistent SCF convergence for all studied systems (perfect and defective). For the perfect BLCO, it also provided results which closely agreed with the theoretical findings of Maria *et al.*^11^ and experimental measurements. We note that the *U* parameters employed in this work correspond to values typically derived from linear response calculations.^30^ A sensitivity analysis performed (see SI, Table S4) indicates that varying the *U* parameters within a physically reasonable range does not alter the overall picture, affecting the properties of the perfect BLCO only quantitatively.

### 3.3 Perfect BLCO and methodology validation

The chosen theoretical approach was validated by considering perfect BLCO and investigating its equation of state and all related properties. For this purpose we performed a series of 11 single point calculations, which differed in the supercell volume. We considered ±5% volume deformations, with 1% step. Based on that we constructed the *E*(*V*) curve which was later fitted with the Birch–Murnaghan equation of state,^31^ obtaining all of its parameters: the bulk modulus *B* and its pressure derivative *B*′, the equilibrium volume *V*_0_ and energy *E*_0_.

From the *V*_0_ parameter we determined the equilibrium lattice parameter *a*_0_. The obtained value of *E*_0_ was used to calculate the cohesive energy (from individual atoms). It was found from the equation:^32^

9 *E*_coh_ = *E*^uc^_0_ − 0.5*E*_Ba_ − 0.5*E*_La_ − *E*_Co_ − 3*E*_O_

Here, *E*^uc^_0_ = *E*_0_/8 represents the total energy of a single unit cell of the considered perovskite, while *E*_Ba_, *E*_La_, *E*_Co_, and *E*_O_ are the total energies of the atoms in their ground states. These energies were obtained from separate DFT calculations, considering isolated atoms. The calculated cohesive energy was compared against the experiment.

The above procedure was performed for the standard DFT and DFT + *U* methodologies, while all the remaining calculations had the same setup. The purpose here was to evaluate how the incorporation of the +*U* corrections affects the predicted properties of BLCO. In addition, we also checked how the predicted band structures and density of states differs in these two methodologies. These calculations were done using the equilibrium lattice parameters *a*_0_, obtained from the EOS.

### 3.4 Calculations for defects in BLCO

Having validated the methodology, we moved to the calculations for defects. As mentioned earlier, we considered three types of defects: single oxygen vacancy, double oxygen vacancy forming *trans*-O pair, and a complex defect, *i.e.* oxygen–cobalt–oxygen vacancy. To study these defects, we removed – respectively – 1, 2, and 3 atoms from the 2 × 2 × 2 supercell and performed geometry relaxation of such obtained systems, using DFT + *U*.

Prior to that, the total charge of the supercell was adjusted such that it matched charges predicted by eqn (2)–(7), *i.e.* 0 and 2+ for single oxygen vacancy, 0 and 4+ for double oxygen vacancy (*trans*-O vacancy pair), 0 and 1+ for complex oxygen–cobalt–oxygen vacancy. In total, we considered six defective supercells, studying each defect in its charged and neutral states. We note that the studied supercells formally correspond to the following compositions of the BLCO compound: Ba_0.5_La_0.5_CoO_3−*δ*_ with *δ* = 0.125 and 0.25 for single and double oxygen vacancy, respectively. The formula Ba_0.5_La_0.5_Co_1−*γ*_O_3−*δ*_, with *δ* = 0.25 and *γ* = 0.125, describes the supercell with the complex oxygen–cobalt–oxygen vacancy.

The geometry relaxation was carried out using the Broyden–Fletcher–Goldfarb–Shanno (BFGS) method. The dimensions of the supercell were also optimized. The relaxation was considered converged when the following four criteria were all met at the same time: d*E*/ion < 10^−5^ eV, *F*_max_ < 0.03 eV Å^−1^, d*R*_max_ < 0.003 Å, and *S*_max_ < 0.03 GPa. The four symbols respectively represent: change in the total energy between two consecutive iterations, maximum force acting on any ion, maximum observed ionic displacement between two consecutive iterations, and the maximum component of the stress tensor. Their application allowed us to find well-converged defect structures. The obtained relaxed structures were later analyzed from various perspectives.

## 4 Results and analysis

### 4.1 Perfect BLCO – methodology validation

#### 4.1.1 Cohesive energy and equation of state

As the first step of validating the developed description method, the results given in Table 1 represent the obtained cohesive energies, calculated based on eqn (9). The experimental synthesis of BLCO is a two-step thermodynamic process that occurs at temperatures of 1100 °C for solid-state sintering or at 25 °C when precipitation method is used.^1,3^ The first step is endothermic and the increase in the temperature of the reactant elements is essential for overcoming the reaction activation barrier. At elevated energy, the formation of BLCO occurs and the negative value of *E*_coh_ implies that it is an exothermic reaction with a resultant thermodynamically stable perovskite. In Table 1, the cohesive energy obtained with the DFT + *U* method has a value of −22.9516 eV which is relatively close to the experimental value of −22.1931 eV (ref. 33) of the oxygen deficient BLCO. This signifies that the DFT + *U* method is reliable for describing the perfect BLCO. On the other hand, the standard DFT fails in the prediction of *E*_coh_, providing a result which differs by approximately 4.4 eV from the experimental value.

**Table 1:** The cohesive energy (*E*_coh_) of the perfect BLCO. The experimental value corresponds to oxygen non-stoichiometry (*δ* > 0) at 25 °C

	*E*_coh_ (eV)
Standard DFT	−26.6359
DFT + *U*	−22.9516
Experiment^33^	−22.1931

**Fig. 3:**
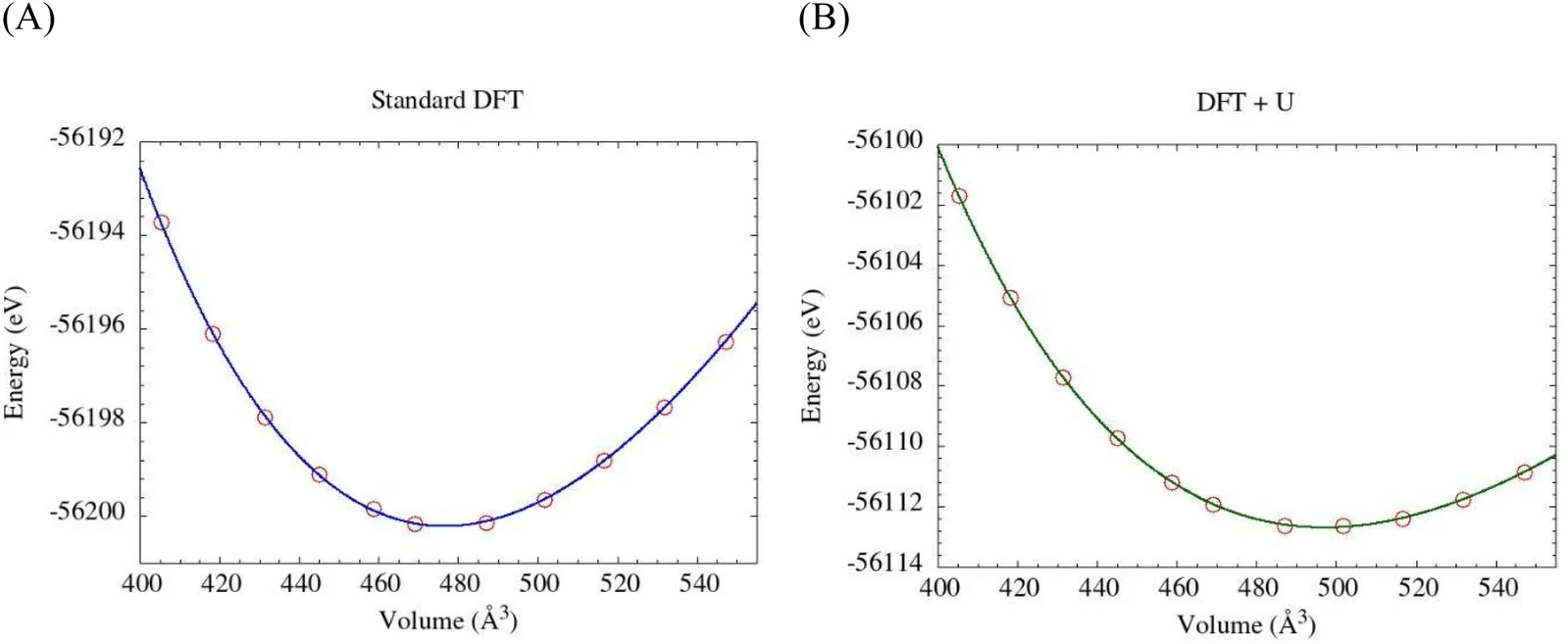
Equation of state of the perfect BLCO as predicted with the standard DFT (panel A) and DFT + *U* (panel B). The points represent calculation results while the solid lines are the fitted Birch–Murnaghan equations of state.

In the second step, the energetics of the perfect BLCO was investigated based on the equation of state calculations. The *U*-shaped character of the *E*(*V*) dependencies obtained (Fig. 3) shows that both methods, *i.e.* standard DFT and DFT + *U*, predict BLCO as a stable compound over the ±5% volume deformations.

By fitting the energy *versus* volume dependencies with the Birch–Murnaghan equation of state, we obtained the structural and mechanical properties of BLCO, which are in Table 2. Both methods slightly overestimate the lattice parameter *a*_0_, as compared to the experiment. However, the experimental value from Table 2 is for non-stoichiometric BLCO (with *δ* > 0) and the presence of oxygen vacancies can be hypothesized as the cause for differences seen in the equilibrium volume. We also note that DFT calculations typically overestimate lattice parameters.

**Table 2:** Structural and mechanical properties of the perfect BLCO as predicted with the standard DFT and DFT + *U*. The symbols represent: the bulk modulus (*B*) and its pressure derivative (*B*′), the total energy (*E*_0_) and volume (*V*_0_) at the equilibrium. We note that the experimental lattice parameter (*a*_0_) corresponds to the room temperature and oxygen non-stoichiometry (*δ* > 0)

	*B* (GPa)	*B*′	*E*_0_ (eV)	*V*_0_ (Å^3^)	*a*_0_ (Å)
Standard DFT	150.87	3.79	−56200.2	476.527	3.905
DFT + *U*	140.03	4.79	−56112.7	496.678	3.951
Experiment^1^	—	—	—	468.916	3.885

Regarding the mechanical properties, both methods, *i.e.* standard DFT and DFT + *U*, predict similar bulk modulus *B*, differing by only 10 GPa. However, the predicted *B*′ differs significantly. Unfortunately, there is no experimental data with *B* and *B*′ for ferromagnetic BLCO. The only data available in the literature is for anti-ferromagnetic and non-stoichiometric BLCO,^34^ with *δ* = 0.2 and tetragonal structure. It reported a considerably higher *B* = 235 (5) GPa, which cannot be compared to our result because of the differences in the structure. In the tetragonal BLCO, the absence of the CoO_6_ octahedra tilts results in no angular degrees of freedom, therefore increasing *B*.^34^ It is worth mentioning that the same study reported 146 GPa bulk modulus for Sr_0.3_La_0.7_CoO_3_, which is structurally similar to the perfect BLCO, and this supports our findings.

#### 4.1.2 Electronic structure

**Fig. 4:**
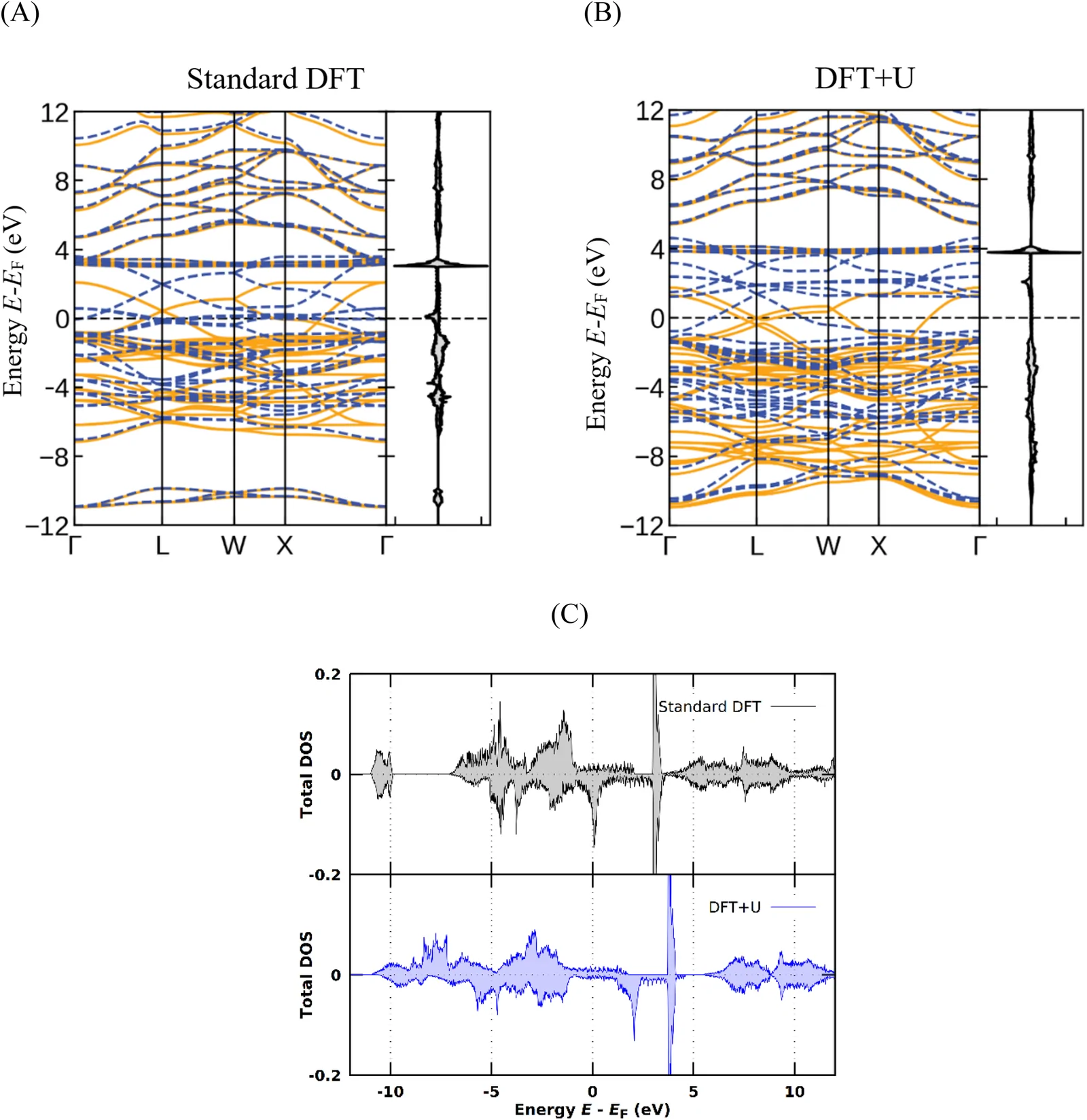
The band structure and total density of states (TDOS) of the perfect BLCO as predicted with the standard DFT (panel A) and DFT + *U* (panel B). In panels A and B, the yellow and blue lines represent the up- and down-spin channel, respectively. Panel C is a magnified comparison of TDOSs. Here and in all later figures, density of states is expressed in arbitrary units, and the zero of the energy corresponds to the Fermi energy *E*_F_.

Now we turn to the discussion of BLCO's electronic structure. In Fig. 4 we present the band structure diagrams and density of states (DOS) plots, comparing results obtained with the standard DFT and DFT + *U*. In both cases, no bandgap was observed in the down-spin channel. However, a band gap was observed in the up-spin channel. This implies that the perfect BLCO in the ferromagnetic state exhibits spin-dependent behavior and is semi-metallic.^11,35,36^ The down-spin channel describes its conductive character while the up-spin channel is responsible for its insulating properties. The experimental electronic band gap of the half-metallic BLCO is 0.03 eV.^20^

The ferromagnetic ordering results in an effective magnetic moment, which was determined as 2.67 µ_B_ and 3.0 µ_B_ (per single unit cell) based on the standard DFT and DFT + *U*, respectively. These values are higher than the experimentally measured 2.11 µ_B_^1^, which was, however, measured for 198 (3) K, while our results correspond to the absolute zero temperature. Notably, a definite saturation magnetic moment from experiment is unattainable because the disordered BLCO strongly depends on the oxygen non-stoichiometry, the ratio of Co^3+^/Co^4+^, the Co spin state (low, intermediate & high spin state), temperature and applied magnetic field.^1^ The effective magnetic moment is mostly due to the Co atoms, which on average introduce 2.16 µ_B_ (standard DFT) or 2.96 µ_B_ (DFT + *U*). These Co magnetic moments are higher than 1.76 µ_B_ reported by Maria *et al.*^11^ based on FP-LAPW + lo calculations, however, these differences may originate from how the local magnetic moments were calculated.

**Fig. 5:**
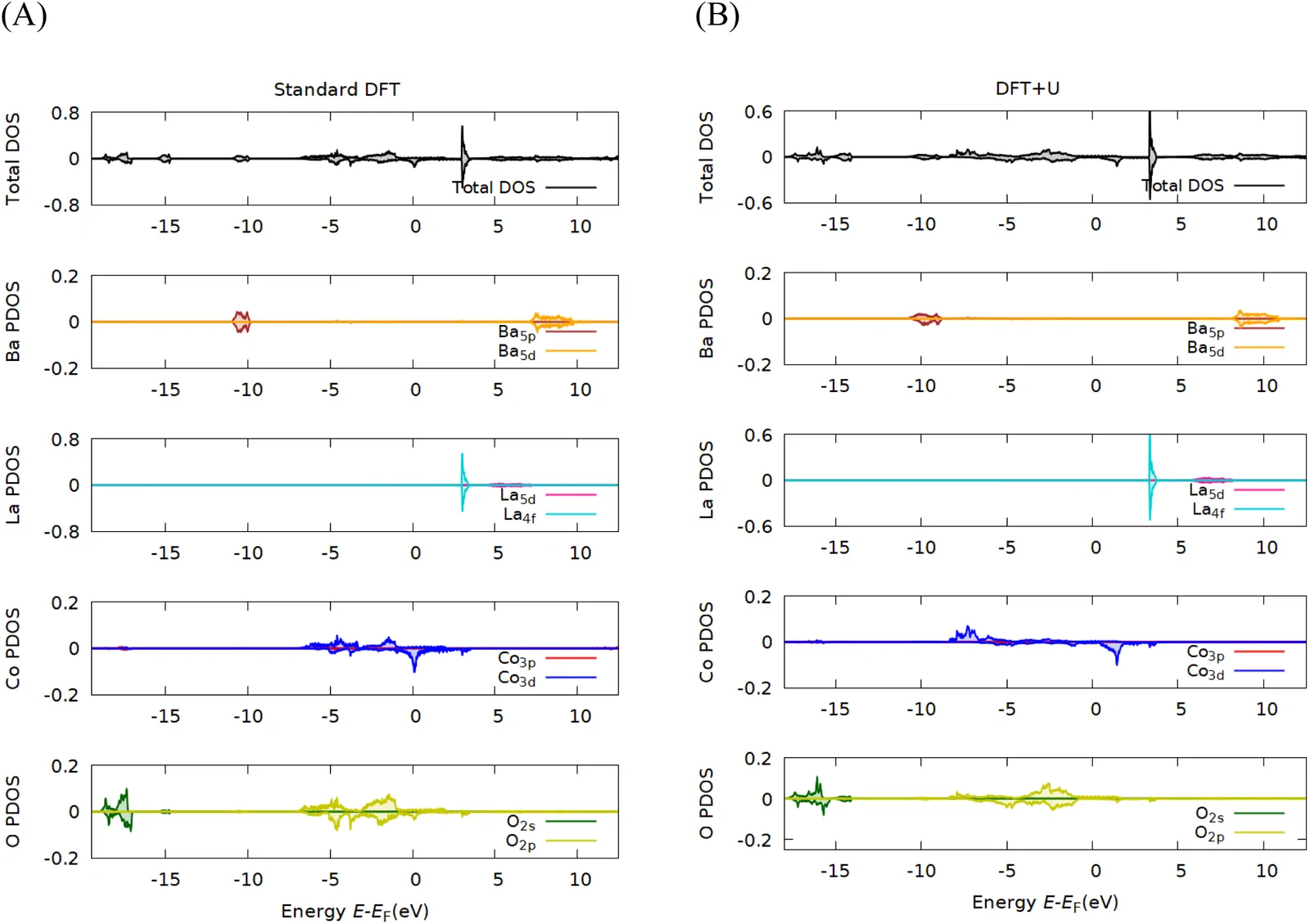
The total density of states of the perfect BLCO and its decomposition into partial densities of states. Two panels compare results obtained with the standard DFT (panel A) and DFT + *U* (panel B).

As seen in Fig. 5, in the BLCO the overlapping valence and conduction bands are formed from O 2p and Co 3d states. Their hybridization forms a continuum of states that span the energy range from nearly −7.5 eV to +4.2 eV in the standard DFT, and from −10 eV to +4.6 eV in the DFT + *U*. This difference in the strength of Co–O hybridization reflects itself in the up-spin band gap, where DFT + *U* predicts a value of 1.88 eV, which is 1 eV higher than 0.83 eV obtained from standard DFT.

Above the partially filled Co 3d conduction band, there is another well visible feature attributed to the La 4f states. It positions itself at +4.3 eV (standard DFT) or +4.6 eV (DFT + *U*). The narrowness of the La 4f band leads to an intense absorption and emission in optical measurements.^37^ Because of its strong spatial localization, the electrons from La 4f band are unlikely to participate in electrical conductivity. They are also chemically less reactive and will unlikely form chemical bonds, as compared to the delocalized La 5d electrons.^37^

The La 5d state placements range from +5.1 eV to +6.7 eV in standard DFT, and from +6 eV to +7.8 eV in DFT + *U*. Above the La 5d states are the Ba 5d states, being positioned between +7.5 eV and +9.6 eV in standard DFT, and between +8.5 eV and 10.5 eV in DFT + *U*. Both the La 5d and Ba 5d states form the higher conduction bands.

Below, the O 2p valence band are lower valence bands. The first one has a Ba 5p character and is located between the energies from −10.8 eV to −10 eV (standard DFT), and from −10.5 eV to −9 eV (DFT + *U*). The lower lying O 2 s states extend between −19 eV to −14.8 eV in standard DFT), and between −17.5 eV and −14.2 eV in DFT + *U*. The positioning of semi-core states, lying deeper in the energy, are presented in Table 3.

**Table 3:** Positioning of the semi-core states in the perfect BLCO. A single value (two values) indicates the presence of a single narrow peak (or two narrow peaks). If range is given, then the corresponding DOS feature is broad, extending between given values

State	Standard DFT		DFT + *U*	
	*E* − *E*_F_ (eV)		*E* − *E*_F_ (eV)	
	Up-spin	Down-spin	Up-spin	Down-spin
Co 3s	−98.2	−94.8	−97.3	−94.6
Co 3p	−62.3, −61.1	−58.2, −57.9	−60.5	−58.0
La 5s	−31.4 to −31.2		−31.6 to −31.5	
Ba 5s	−25.0 to −24.6		−24.0 to −23.8	

As shown in Table 3, there are minimal differences seen in the positioning of the Co 3s, La 5s and Ba 5s semi-core states, when comparing the standard DFT and DFT + *U* predictions. However, applying the +*U* correction alters both the positioning and number of peaks in the case of Co 3p state. In standard DFT, the Co 3p state is split, with the distance between its peaks being 1.2 eV (up-spin) and 0.3 eV (down-spin). In DFT + *U*, the two peaks collapse in both spin channels. We attribute this change to exchange interactions, which also increase local magnetic moments of Co atoms in DFT + *U*.

The latter is well visible in the Mulliken population analysis, which showed that in the standard DFT each Co atom has a spin of 2, while DFT + *U* predicted spin of 3. We note that both methods predicted the electron configuration of Co atoms to be nearly 3d.^7^ However, in DFT + *U* the occupations of up- and down-spin channels were 4.8 and 2.0, respectively; while in standard DFT they were 4.6 and 2.5. This results in a higher magnetic moment in DFT + *U*. The highlighted differences in occupations and magnetic moments reflect in the lower lying states, mostly affecting the Co 3p semi-core state.

In addition, the carried out population analysis showed that in both methods all Co atoms are equivalent, having the same chemical environment and electron structure. This means that our calculations predict that there are no two distinct (+3 and +4) oxidation states of Co atoms in the perfect BLCO. Instead, the predicted Co oxidation state appears to be somewhere between +3 and +4. This result agrees with the experimentally measured +3.5 Co valency.^1^ Notably, our computational result contradicts the predictions of the point defect chemistry model (see eqn (1)), which assigns two distinct oxidation states to Co atoms.

**Fig. 6:**
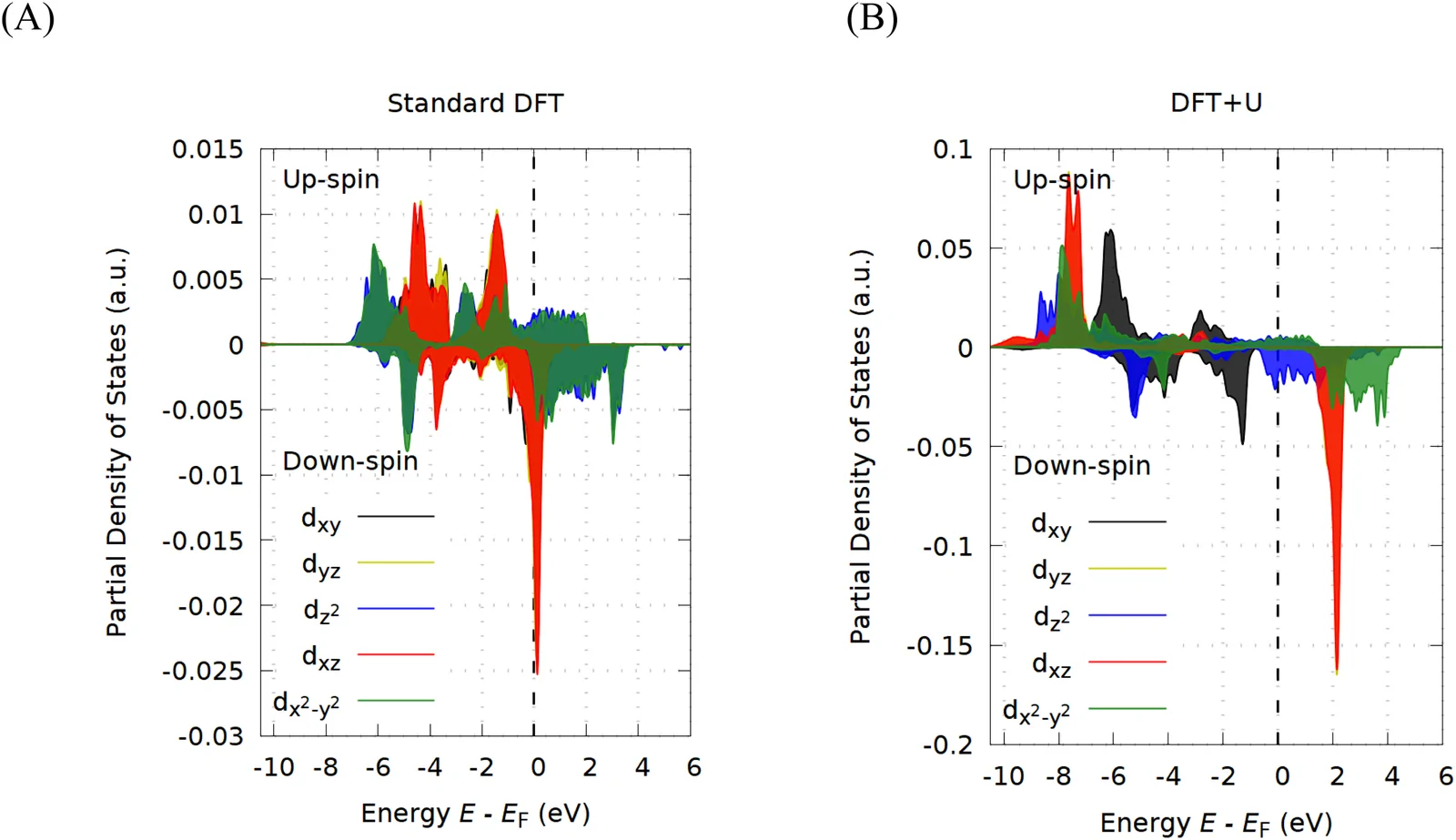
The decomposition of Co 3d PDOS into contributions from t_2g_ (d_*XY*_, d_*XZ*_ and d_*YZ*_) and e_g_ (d_*X*^2^−*Y*^2^_ and d_Z^2^_)levels. Two panels compare results obtained with the standard DFT (panel A) and DFT + *U* (panel B).

The analysis of Co 3d states occupancies showed significant differences between the standard DFT and DFT + *U* results. In standard DFT, the up-spin channel of all three Co 3d – t_2g_ states (d_*XY*_, d_*XZ*_ and d_*YZ*_) are almost fully occupied with occupations reaching 1, whereas the Co 3d – e_g_ states (d_*X*^2^−*Y*^2^_ and d_Z^2^_) are partially occupied, with both occupations being 0.79. In the down-spin channel, all three Co 3d – t_2g_ states and two Co 3d – e_g_ are partially occupied, with the corresponding occupations being 0.64 (t_2g_) and 0.34 (e_g_). These differences in the occupations of up- and down-spin Co 3d states reflect in their positioning on the energy scale, as seen in Fig. 6A.

DFT + *U* also predicts that in the up-spin channel, the Co 3d – t_2g_ states (d_*XY*_, d_*XZ*_ and d_*YZ*_) are nearly fully occupied. However, it predicts higher (*i.e.* 0.93) occupation of the e_g_ states (d_*X*^2^−*Y*^2^_ and d_Z^2^_) in the up-spin channel. Notable changes are observed in the down-spin channel, where the t_2g_ – d_*XY*_ is almost fully occupied, while two remaining t_2g_ states (d_*YZ*_ and d_*XZ*_) are nearly empty, with occupations being 0.07. In addition, the Co 3d – e_g_ states d_Z^2^_ and d_*X*^2^−*Y*^2^_ have occupancies of 0.53 and 0.22, respectively. These changes are reflected in the energy positioning of Co 3d states, leading to an increased intensity of features. The comparison of partial densities of state (PDOS, see Fig. 6) shows that in DFT + *U*, the intensity of most pronounced peaks is about 10- and 3-times higher than in standard DFT, in the up- and down-spin channels, respectively.

### 4.2 Defects in BLCO

#### 4.2.1 Structural relaxation

**Fig. 7:**
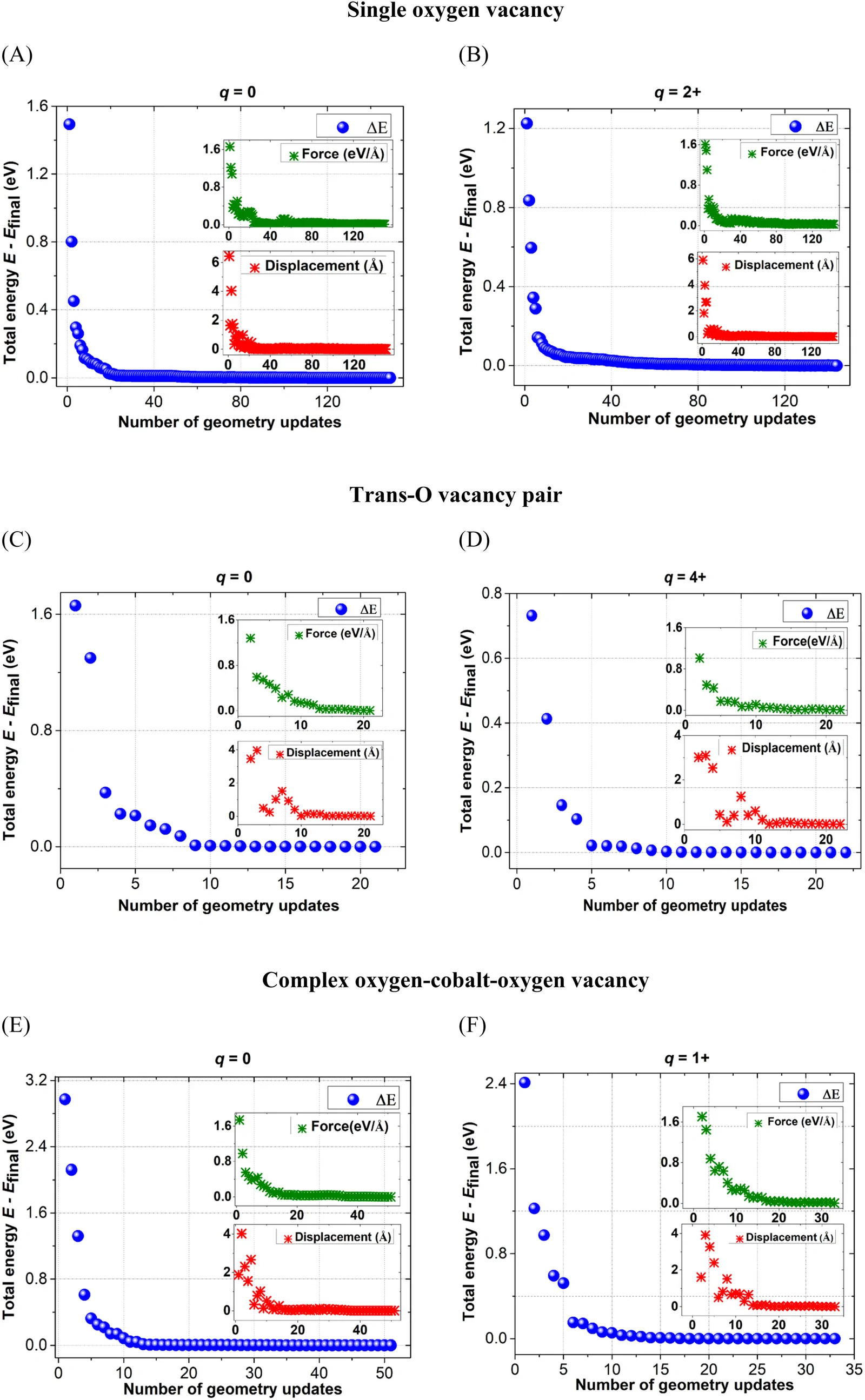
Evolutions of the total energy, forces and displacements in the carried out geometry relaxations. Note that the total energy is measured with respect to its converged value (*E*_final_).

We start the discussion of defective systems with a brief description of the carried out energy minimizations. In all six cases, we were able to find well-converged relaxed structures of defects. As seen in Fig. 7, finding the energy minimum required from 20 (*trans*-O vacancy pair), through 30–50 (complex defect), up to 130 (single oxygen vacancy) BFGS iterations. Despite the comparable number of geometry updates observed for the neutral (*q* = 0) and charged (*q* > 0) supercells, the relaxations done for the neutral defects took longer, because they required on average two-three times more SCF cycles in each step of geometry optimization.

We note that we also considered different charge states, like *q* = 1+ for a single oxygen vacancy. However, in this calculation (and all other not reported calculations) we observed problems with the convergence of the SCF procedure, which was trapped after several SCF steps, displaying oscillatory behaviour. The additional analyses that we performed showed that a specific electron state was produced close to the Fermi energy. In the subsequent SCF steps this state was constantly flipping, locating itself in various voids between the CoO_6_ octahedra, and consequently preventing convergence. From the physical perspective, the observation of this state suggests that a single oxygen vacancy with *q* = 1+ charge is unstable in BLCO.^38,39^

Table 4 summarizes all carried out relaxations, comparing the essential results for all studied defects and showing their influence on the magnetic properties and structural parameters of BLCO. Later on, we will refer to this table in our detailed discussions, presented separately for each considered defect in three subsequent subsections.

**Table 4:** Influence of defects on the magnetic and structural properties of BLCO. All results correspond to DFT + *U* calculations. The first row presents data for the perfect BLCO, which serves as a reference. The local Co magnetic moment was obtained as an average over all Co atoms and presents the magnetic moment of a single 5-atom unit cell

System/defect type	Defect charge *q*	Local Co magnetic moment *µ*_Co_ (*µ*_B_)	Effective magnetic moment *µ*_eff_ (*µ*_B_)	Supercell volume *V* (Å^3^)	Supercell dimensions (Å)	Cell angles (degrees)
Perfect BLCO	N/A	2.96	3.00	496.678	*a* = 7.964001	*∝* = 90.0001
					*b* = 7.964001	*β* = 89.9999
					*c* = 7.836636	*γ* = 89.9998
Single oxygen vacancy	2+	2.74	2.45	494.683	*a* = 7.953985	*∝* = 90.0999
					*b* = 7.937593	*β* = 90.0265
					*c* = 7.835276	*γ* = 89.9238
	0	2.96	2.97	513.756	*a* = 8.076970	*∝* = 89.9405
					*b* = 8.006512	*β* = 90.2068
					*c* = 7.944541	*γ* = 89.8945
*Trans*-O vacancy pair	4+	2.73	2.50	468.138	*a* = 7.747950	*∝* = 89.9995
					*b* = 7.870606	*β* = 89.9988
					*c* = 7.676779	*γ* = 89.9998
	0	3.07	3.36	516.906	*a* = 7.991637	*∝* = 90.0683
					*b* = 8.175186	*β* = 90.0314
					*c* = 7.911854	*γ* = 89.9965
Complex oxygen–cobalt–oxygen vacancy	1+	2.95	2.28	511.394	*a* = 7.898591	*∝* = 90.0133
					*b* = 8.115206	*β* = 90.0039
					*c* = 7.978236	*γ* = 90.0003
	0	2.91	2.37	533.928	*a* = 7.926690	*∝* = 90.5453
					*b* = 8.160341	*β* = 88.9734
					*c* = 8.256046	*γ* = 89.9552

#### 4.2.2 Single oxygen vacancy

As stated in the theoretical review, each oxygen vacancy becomes an electron donor and may have a zero or positive charge, as described earlier in eqn (2) and (3), respectively. In our case, defective supercells with charges *q* = 0 and 2+ model these situations. However, our results showed that the introduction of an oxygen vacancy leads to a charge redistribution, which is different from what defect chemistry predicts. According to it, the introduction of oxygen vacancy should result in either charge localization (*q* = 0, see eqn (2)) or delocalization (*q* = 2+, eqn (3)). In contrast, our DFT + *U* calculations showed that both phenomena occur at the same time for *q* = 0 and 2+, but to varying degrees. In what follows, we will illustrate these effects, also showing in detail how the introduction of oxygen vacancy affects the electronic structure and various properties of BLCO.

**Fig. 8:**
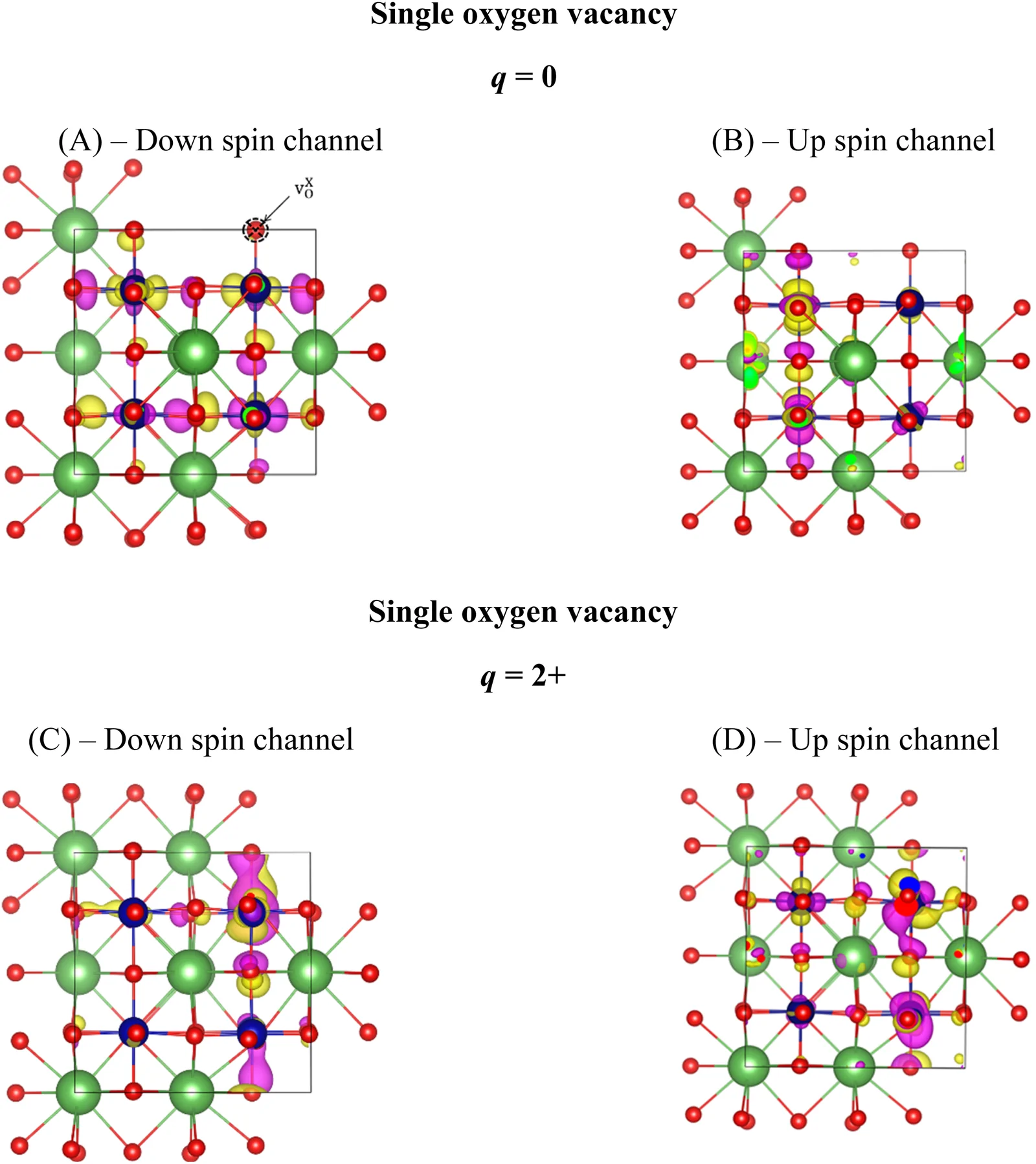
Representative Kohn–Sham spin-orbitals *φ*_KS_ in BLCO containing a single oxygen vacancy. The presented orbitals correspond to electron levels closest to *E*_F_. Isosurfaces of |*φ*_KS_|^2^ = 10^−2^ e/Å^3^ are shown, with yellow and violet colors distinguishing the sign of *φ*_KS_. The coloring of atoms is the same as in Fig. 1. The position of the single oxygen vacancy (indicated in panel A) is the same in all panels.

Fig. 8 is a visual representation of these effects, presenting selected Kohn–Sham spin-orbitals. In the presented cases, the delocalization is well-visible in the case of the neutral vacancy (*q* = 0), while the localization is more pronounced in the case of charged oxygen vacancy (*q* = 2+). The occurrence of these effects is a result of two charge redistribution mechanisms, occurring simultaneously after atom removal.

This charge may be redistributed by populating O 2p – Co 3d conduction band, which is partially filled in the half-metallic BLCO. This process is well-visible in Fig. 8A and B, where the lobes of *φ*_KS_ are centered around Co and O atoms, the *φ*_KS_ state expands over the entire system and condensates around CoO planes. As a result, it produces a typical conduction state and implies charge delocalization.

The second mechanism originates from a tendency of selected Co atoms to undergo reduction. It is well-visible in Fig. 8C and D, where the lobes of *φ*_KS_ are mostly centered around selected Co atoms closest to the single oxygen vacancy. Since the *φ*_KS_ state is bounded spatially, the second mechanism leads to a charge localization.

The above analysis may suggest that charge delocalization and localization are specific for the neutral and charged system, respectively. However, we note that the visualizations from Fig. 8 are just for illustration purposes and that both effects occur simultaneously for *q* = 0 and *q* = 2+.

The additional proof is presented in Table S1, which summarizes the carried out population analysis, showing the changes in atomic charges and spins of Co and O atoms, as well as changes in the Co–O bond lengths and occupations. These changes were measured with respect to the perfect BLCO. Therefore, the reported values reflect the overall changes, accounting for both the redistribution of the excess charge remaining after defect formation (applicable to the neutral vacancy, with *q* = 0) and various other charge redistribution processes triggered by the introduction of the defect. For each considered quantity, various statistical metrics are presented, like minimum, maximum, and mean values. The numbers of atoms and bonds displaying significant changes, greater than the specified thresholds, are also given.

As seen from Table S1, for *q* = 2+ a total of 17 Co and O atoms participate in the charge redistribution, displaying significant change in charge or spin, while for *q* = 0, there are 11 such atoms. In addition, in *q* = 2+ there are twenty four more Co–O bonds than in *q* = 0 that were notably affected by the introduction of a defect. The quoted numbers support our earlier statements, also showing that for *q* = 2+ the degree of delocalization is higher.

What is also important, the charges of the Co2 and Co7 atoms, which are nearest to the defect, changed by −0.141 (*q* = 2+) and −0.168 (*q* = 0). This shows that only a mild reduction of these atoms occurred. This observation is contrary to defect chemistry, which predicts strong Co reduction.

The introduction of a single oxygen vacancy and the corresponding charge redistribution has also an influence on the crystal lattice. As seen in Fig. 8, mild distortions of the CoO_6_ octahedra occur and they are well-visible in the changes of bond lengths, specifically those connecting Co and O. In Table S1 we provide information on these changes. It shows a non-uniform elongation of Co–O bonds for *q* = 0, which results in an anisotropic lattice expansion. Contrary to that, for *q* = 2+ the Co–O bonds contract and this leads to an anisotropic lattice contraction.

The lattice distortions reflect the changes in volume. Our results show that the introduction of the neutral oxygen vacancy (*q* = 0) leads to a 3.4% increase in the volume, while charged oxygen vacancy (*q* = 2+) causes volume decrease, by nearly 0.4% relative to the perfect BLCO.

**Fig. 9:**
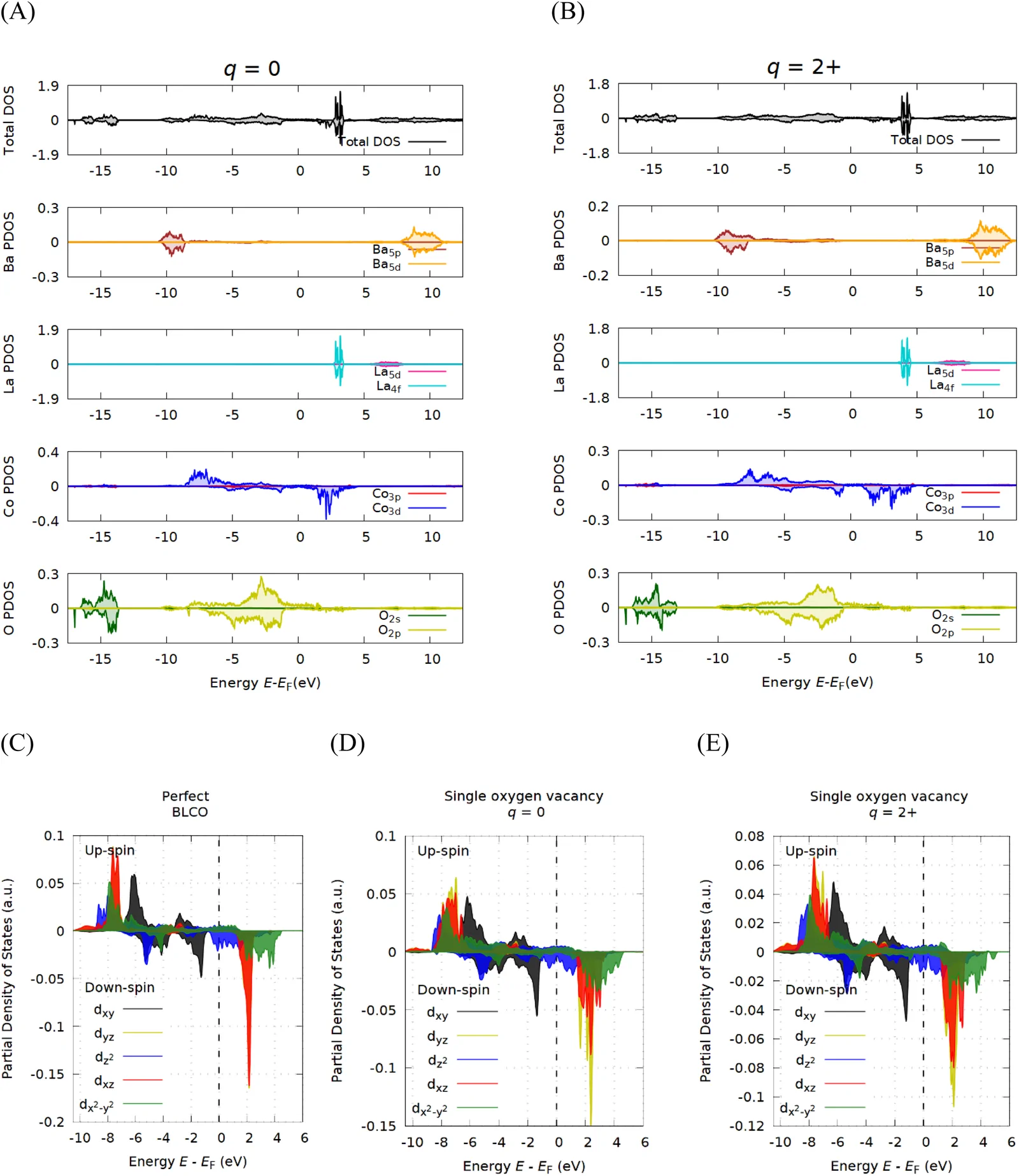
Influence of the single oxygen vacancy on the electronic structure of the BLCO. Panels A and B present TDOS and its decomposition into PDOS, comparing the results obtained for *q* = 0 and *q* = 2+. Panels C–E present the PDOS of the Co 3d states, comparing the results obtained for the defective systems (D and E) with the perfect BLCO (C).

Now we turn to a discussion on how the introduction of a single oxygen vacancy reflects in the band structure. For this purpose we will use DOS diagrams presented in Fig. 9. In what follows, we will highlight only the most notable changes. However, for the sake of completeness, in the supplementary material (see Table S2) we provide full information on the positioning of all features visible on DOS.

The introduction of a single oxygen vacancy with *q* = 0 leads to the splitting of the Co 3s semi-core state into three distinct peaks in both spin channels, while in the perfect BLCO only a single peak was present. In addition, the Co 3p peak splits into five-six features. The same two phenomena are observed for *q* = 2+. For the neutral single oxygen vacancy, the peaks corresponding to the La 5s, Ba 5s, O 2s and Ba 5p states span similar energy ranges as in the perfect BLCO. However, charging the oxygen vacancy (*q* = 2+) causes broadening of these features and shifts them toward *E*_F_. The most notable change is observed for the Ba 5p state which spans energies from −10.1 eV to −7.2 eV in the defective system, while in the perfect BLCO it spans energies from −10.5 eV to −9.0 eV.

In the systems containing an oxygen vacancy, the Co 3d – O 2p bands occupy similar positions as in perfect BLCO. However, for *q* = 0 the width of the O 2p band decreases by an estimate of 0.8 eV, while charging the defect (*q* = 2+) causes a 1.3 eV increase in the width of the Co 3d band. In addition, the La 4f states move closer to *E*_F_ in the defective systems, by approximately 1.6 eV and 0.6 eV for *q* = 0 and 2+, respectively. The La 4f peak also broadens and splits, with its maxima being separated by 0.3 eV (*q* = 0) and 0.5 eV (*q* = 2+).

The introduction of the neutral oxygen vacancy does not considerably affect the La 5d conduction band, whereas charging the defect (*q* = 2+) causes its slight shift to higher energies. The presence of the neutral single oxygen vacancy also induces a minor negative shift of the Ba 5d conduction band, while charging the defect shifts this feature upwards.

In the defective systems, there is a reduction in the Co 3d – O 2p hybridization due to the removal of two Co–O ligands and the disruption in the Co–O–Co connectivity. This may affect both energy positioning of the Co 3d states and how they are populated. Compared to the perfect BLCO, the presence of an oxygen vacancy leads to a decrease in the intensity of the Co 3d e_g_ and t_2g_ peaks as shown in Fig. 9D and E. However, for their up-spin channels, the calculated average occupancies (approximately 1) of the Co 3d – t_2g_ states (d_*XY*_, d_*XZ*_ and d_*YZ*_) in defective systems are the same as in the perfect BLCO (see Table S3). Similarly, no considerable changes are visible in the occupations of the Co 3d – e_g_ states (d_*X*^2^−*Y*^2^_ and d_Z^2^_). This points to negligible changes in the band occupancy of the up-spin channel.

In the down-spin channel, the average occupancy of the Co 3d t_2g_ – d_*XY*_ states is also the same as in the perfect BLCO (around 1), while for the d_*YZ*_ and d_*XZ*_ states some minor (±0.02) changes in average occupancies are observed. Regarding the Co 3d – e_g_ states (d_Z^2^_ and d_*X*^2^−*Y*^2^_) in the defective systems, these are also partially occupied with average occupancies being 0.60 and 0.24 for *q* = 0, respectively. For *q* = 2+ we obtained 0.57 and 0.26, respectively. Therefore, in both cases a net increase (+0.06 for *q* = 0 and 0.04 for *q* = 2+) in the occupation of the Co 3d e_g_ states is observed, as compared to the perfect BLCO. This indicates an increase in population of the down-spin Co 3d band. The observed increase in the occupation of the Co 3d down-spin band contributes to a reduction of the effective magnetic moment in the systems containing oxygen vacancy (see Table 4). However, this reduction in the effective magnetic moment cannot be attributed entirely to Co atoms, as the O atoms also considerably contribute to this effect, as evidenced by the data presented in Table S1.

#### 4.2.3 *Trans*-O vacancy pair

**Fig. 10:**
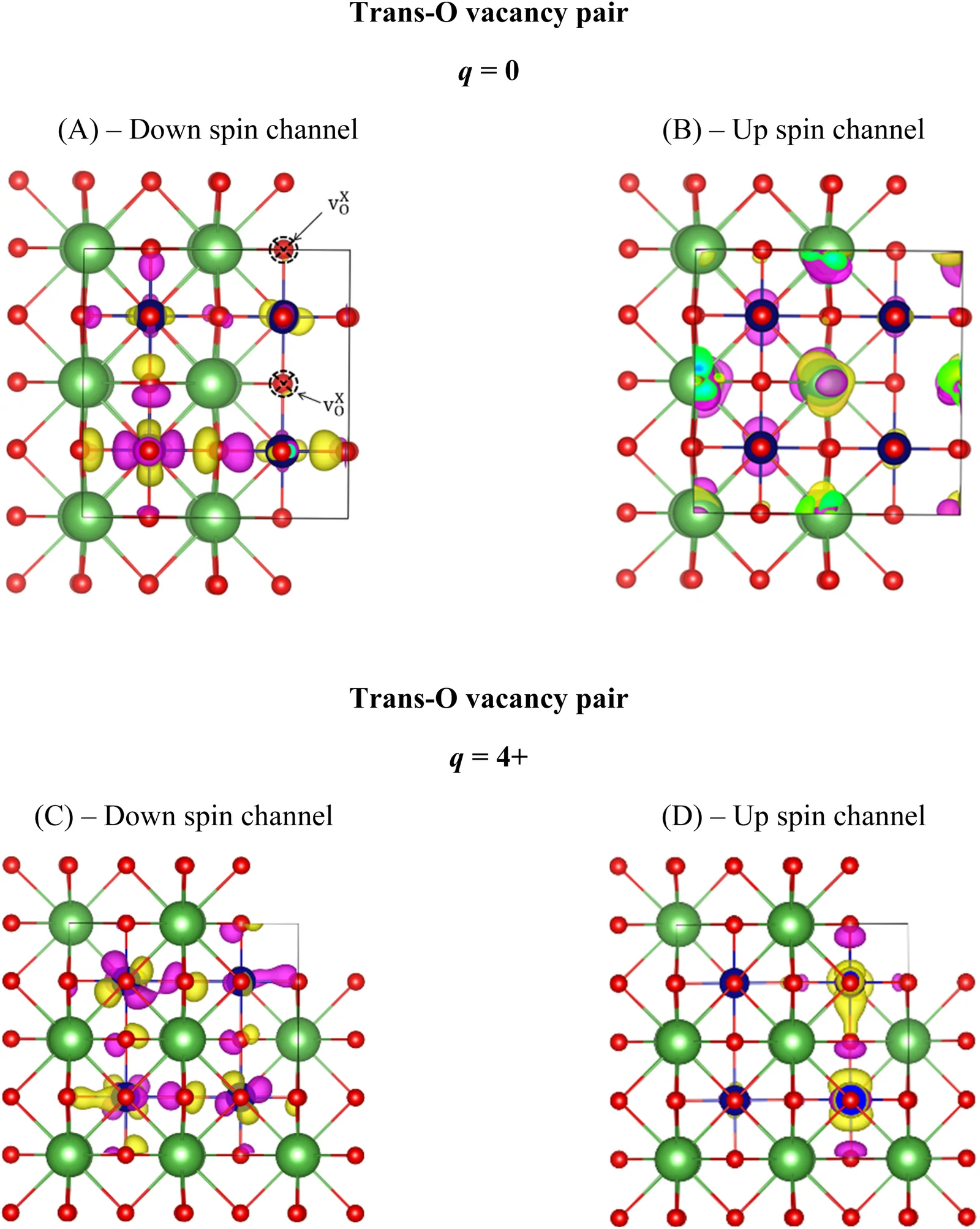
Representative Kohn–Sham spin-orbitals *φ*_KS_ in BLCO containing a *trans*-O vacancy pair. The presentation method is the same as in Fig. 8.

The *trans*-O vacancy pair results presented in this subsection correspond to two oxygen vacancies positioned on the opposite sides of a CoO_6_ octahedron (see Fig. 10A). Defect chemistry predicts that the formation of a *trans*-O vacancy pair may result in either charge localization (*q* = 0, eqn (4)) or delocalization (*q* = 4+, eqn (5)). However, our DFT + *U* results show that both phenomena occur simultaneously to varying degrees. The Kohn–Sham spin orbitals presented in Fig. 10 illustrate these effects.

Here charge delocalization occurs in the down-spin channel and is well-visible in Fig. 10A (*q* = 0) and 10C (*q* = 4+), where the presented states extend over entire CoO planes. This behavior arises because in this defective system the electron populates the Co 3d – O 2p conduction band, which was partially filled in the perfect BLCO. On the contrary, charge localization manifests strongly in the up-spin channel, where the presented states localize around selected atoms. These are Co and La atoms (Fig. 10B, *q* = 0) or only Co atoms (Fig. 10D, *q* = 4+). The observed charge localization in La atoms stems from the fact that in the perfect BLCO the Co 3d – O 2p conduction band is partially filled, therefore the nearby Co atoms provide levels available for populating in defective systems. Once these are filled, the higher lying states provided by the nearby La atoms are also involved for *q* = 0.

The observations made for Fig. 10 are supported by the population analysis (see Table S1), which also indicates that both charge localization and delocalization occur simultaneously for *q* = 0 and *q* = 4+. The localization is visible as significant changes in charges or spins of the individual atoms, while the delocalization manifests as a high number of Co and O atoms involved in the charge redistribution. We found 21 and 18 such atoms for *q* = 0 and *q* = 4+, respectively.

In addition, the analysis of charges shows that two Co atoms which are near the oxygen vacancies (Co2 and Co7) also undergo a mild chemical reduction, with their charges changing by −0.269 for *q* = 0 and −0.099 for *q* = 4+. Thus, the mild Co reduction observed here also negates the defect chemistry prediction of explicit Co reduction as expressed in eqn (4) for *q* = 0.

It is worth mentioning that the introduction of a vacancy pair results in four missing Co–O ligands. This disrupts the potential pathways for electronic transport, as the electrons face higher energy barriers with missing Co–O ligands. Instead, they opt for routes on the CoO planes, which are free of oxygen vacancies. Oxygen vacancies can lead to the release or trapping of electrons.^40,41^ If electrons are released, then a strong reduction of nearby Co atoms is possible as described by eqn (4). The trapping of electrons by oxygen vacancies lead to polaron formation.^40^ It is known that strong polarization as a result of oxygen vacancy formation increases the activation energy of polaron transport.^41^ In our results, we have a mild reduction of Co atoms near the defect locale which points to a mild degree of polarons. Therefore, our result may confer with the experimental observation that in the cobalt-based perovskites an increase in the number of oxygen vacancies leads to a higher polaron activation barrier which is seen as a half-metallic to semi-conducting transition.^42,43^

The introduction of an oxygen vacancy pair considerably distorts the crystal structure, as seen in Fig. 10. For *q* = 0, all of the Co–O bonds undergo expansion, with their length changing by 0.028 Å on average. However, the observed lattice expansion is anisotropic, because of different changes seen in the individual bond lengths. For *q* = 4+, most of the Co–O bonds contract and the mean change in their length is −0.037 Å. This leads to an anisotropic lattice contraction. The observed lattice distortions influence the volume. The introduction of two neutral oxygen vacancies leads to a 4.1% increase in volume, while charging those vacancies results in a 5.7% decrease in volume, relative to the perfect BLCO. Moreover, the supercell containing the charged *trans*-O vacancy pair has a volume of 468.138 Å^3^, which is close to the experimental volume of 468.916 Å^3^, measured for BLCO with the oxygen non-stoichiometry of 0.225.^1^ This non-stoichiometry is close to 0.25 that describes our supercell. This comparison shows that our theoretical description reliably predicts the influence of oxygen vacancies on the volume of BLCO.

**Fig. 11:**
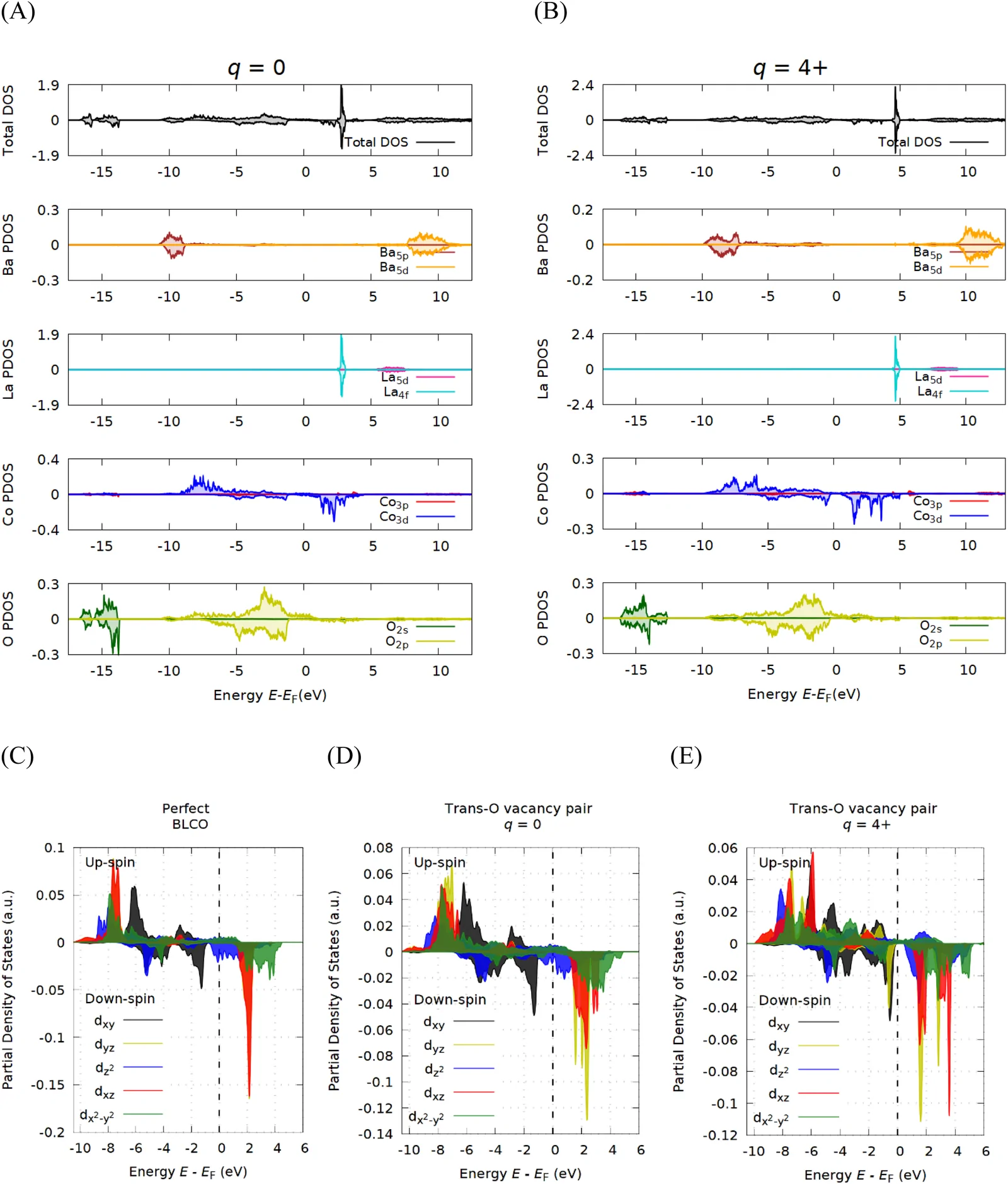
Influence of the *trans*-O vacancy pair on the electronic structure of the BLCO. The presentation method is the same as in Fig. 9.

The presence of a *trans*-O vacancy pair also affects the band structure (see Fig. 11 and Table S2). Similar to the single oxygen vacancy case described earlier, the introduction of two oxygen vacancies leads to a splitting of the Co 3s semi-core states into two-three distinct features in both the up-spin and down-spin channels. For Co 3p states, the splitting phenomenon also occurs, with five–six (*q* = 0) and six–seven (*q* = 4+) peaks being visible on both spin-channels, while there was a single peak in the perfect BLCO. Moreover, the La 5s, Ba 5s, O 2s and Ba 5p states span broader energy ranges than in the perfect BLCO and their positioning shifts towards *E*_F_. Both effects occur for *q* = 0 and *q* = 4+.

In addition, the introduction of the *trans*-O vacancy pair, in both the neutral and charged state, results in a visible (approximately 1 eV) increase in the width of the Co 3d – O 2p conduction band. More importantly for *q* = 4+, a band gap of 0.6 eV appears in the down-spin channel while for *q* = 0 there is no bandgap, same as in the perfect BLCO. This shows that the charged oxygen vacancy pair alters electronic properties of BLCO.

Notably, the La 4f state lying above the conduction band maintains a similar position as in the perfect BLCO for *q* = 4+. Similar to the perfect BLCO, this state produces a single feature on the DOS. The fact that it lacks the splitting observed for the single oxygen vacancy case (see Fig. 9) suggests that the morphology of the La 4f feature may be used in spectroscopy to distinguish the presence of single and double oxygen vacancies. In addition, the *trans*-O vacancy pair with *q* = 0 also produces a single peak for the La 4f state, but it is shifted by −1.8 eV towards *E*_F_. The positions of the higher conduction bands are also slightly altered, with their bottoms experiencing shifts of +0.4 eV (La 5d, *q* = 0), +1.5 eV (La 5d, *q* = 4+), −0.5 eV (Ba 5d, *q* = 0), and +0.5 eV (Ba 5d, *q* = 4+).

We now analyse how the occupation of the Co 3d states change upon the introduction of a *trans*-O vacancy pair. For *q* = 0, the occupations of the Co 3d – t_2g_ states in both spin channels do not change significantly as compared to the perfect BLCO (see Table S3). On the contrary, significant changes are observed for *q* = 4+. They are visible in the up-spin channel, where the occupation of the Co 3d – e_g_ states decreases by 0.07 (d_Z^2^_) and 0.03 (d_*X*^2^−*Y*^2^_) on average. However, the most notable change occurs in the down-spin channel, where the occupation of the Co 3d t_2g_ – d_*YZ*_ state increases by 0.25. In addition, the occupations of the Co 3d – e_g_ states change by +0.13 (d_Z^2^_) and −0.07 (d_*X*^2^−*Y*^2^_). These changes result in an increased splitting of the Co 3d peaks, leading to a decrease in their intensity when compared to the perfect BLCO (compare Fig. 11C and E). They also cause a change in the effective magnetic moment, which for *q* = 4+ decreased to 2.5 µ_B_. However, for *q* = 0 we obtained 3.3 µ_B_ which is higher than in the perfect BLCO, and we attribute this increase to a presence of more unpaired electrons.

#### 4.2.4 Complex oxygen–cobalt–oxygen vacancy

**Fig. 12:**
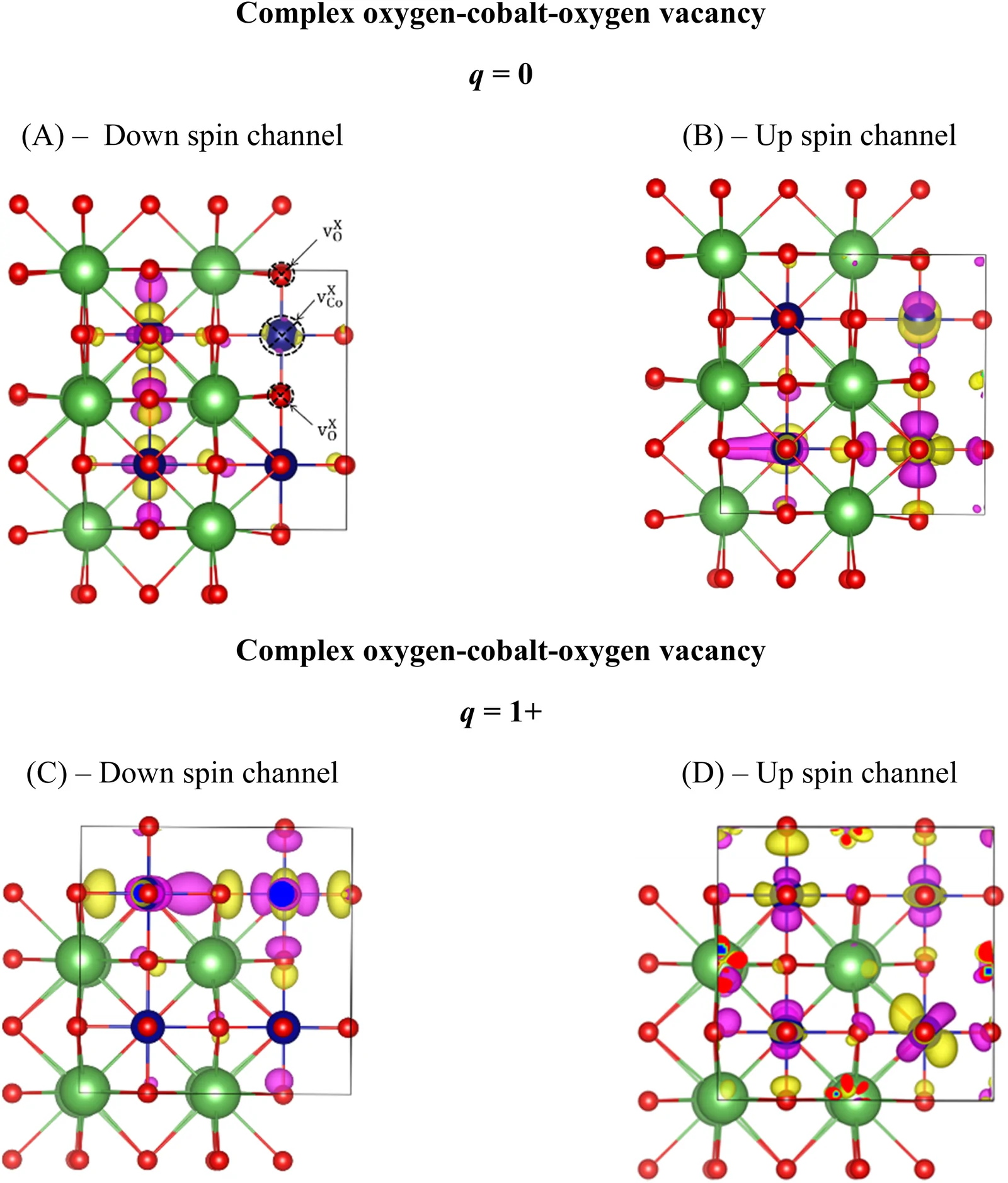
Representative Kohn–Sham spin-orbitals *φ*_KS_ in BLCO containing a complex oxygen–cobalt–oxygen vacancy. The presentation method is the same as in Fig. 8.

Building on the earlier observations made for single and double oxygen vacancies, this subsection discusses the results obtained for the complex defect, formed by removing a cobalt atom and its two neighboring oxygen atoms (see Fig. 12). We recap that defect chemistry predicts three possible scenarios for the formation of this defect, two for *q* = 0 and one for *q* = 1+ (see eqn (6)–(8), respectively). They differ in the charge redistribution, which may occur either in localized (eqn (6)–(8)) or delocalized (also occurs in eqn (7)) manner. However, our DFT + *U* calculations have shown that the charge redistribution proceeds in a more sophisticated fashion, with simultaneous occurrence of charge localization and delocalization.

In a simplified picture provided by the defect chemistry, the reduction of Co sites is considered as a process leading to a charge localization. Instead of strictly localizing the charge on an individual Co site, our results have shown that the BLCO displays a tendency to redistribute the excess charge over many Co and O sites. This corresponds to populating the Co 3d – O 2p conduction band, which is partially filled in the perfect BLCO. This process is well-visible in Fig. 12A (*q* = 0), where the visualized Kohn–Sham orbitals extend over an entire CoO plane, forming a delocalized conduction state. In addition, the tendency to localize charge and reduce Co site is also apparent from this figure, and visible as lobes of *φ*_KS_ centered on the Co atom (Co6) positioned below the cobalt vacancy. Similarly, both effects are also visible in the down-spin channel of *q* = 1+ (Fig. 12C).

The observed charge delocalization manifests in Table S1 as a high number of Co and O atoms and Co–O bonds participating in the charge redistribution. There are 10 (*q* = 0) and 16 (*q* = 1+) such atoms, and 30 (*q* = 0) and 34 (*q* = 1+) such bonds. The simultaneous occurrence of charge localization causes a high change in the charge or spin of the individual atoms. An example is the Co7 atom, which is close to the defect. Its charge changed by −0.148 (*q* = 1+) and −0.273 (*q* = 0), which suggests mild reduction. However, for *q* = 1+ its spin changed more considerably, by −0.514. This shows that in the BLCO the redistribution of charge is more complex and quite different than in the predictions provided by simple defect chemistry models, as it also involves changes in spins. We will comment on this observation in the summary.

The presence of the complex defect causes both elongation and contraction of the Co–O bonds, as evidenced by the minimum and maximum values provided in Table S1. However, most of the Co–O bonds elongate, with the mean change in the bond length being 0.052 Å (*q* = 0) and 0.032 Å (*q* = 1+). The observed anisotropic lattice relaxation leads to an increase in the volume (see Table 4), which increases by 3.0% (*q* = 1+) and 7.5% (*q* = 0), as compared to the perfect BLCO.

**Fig. 13:**
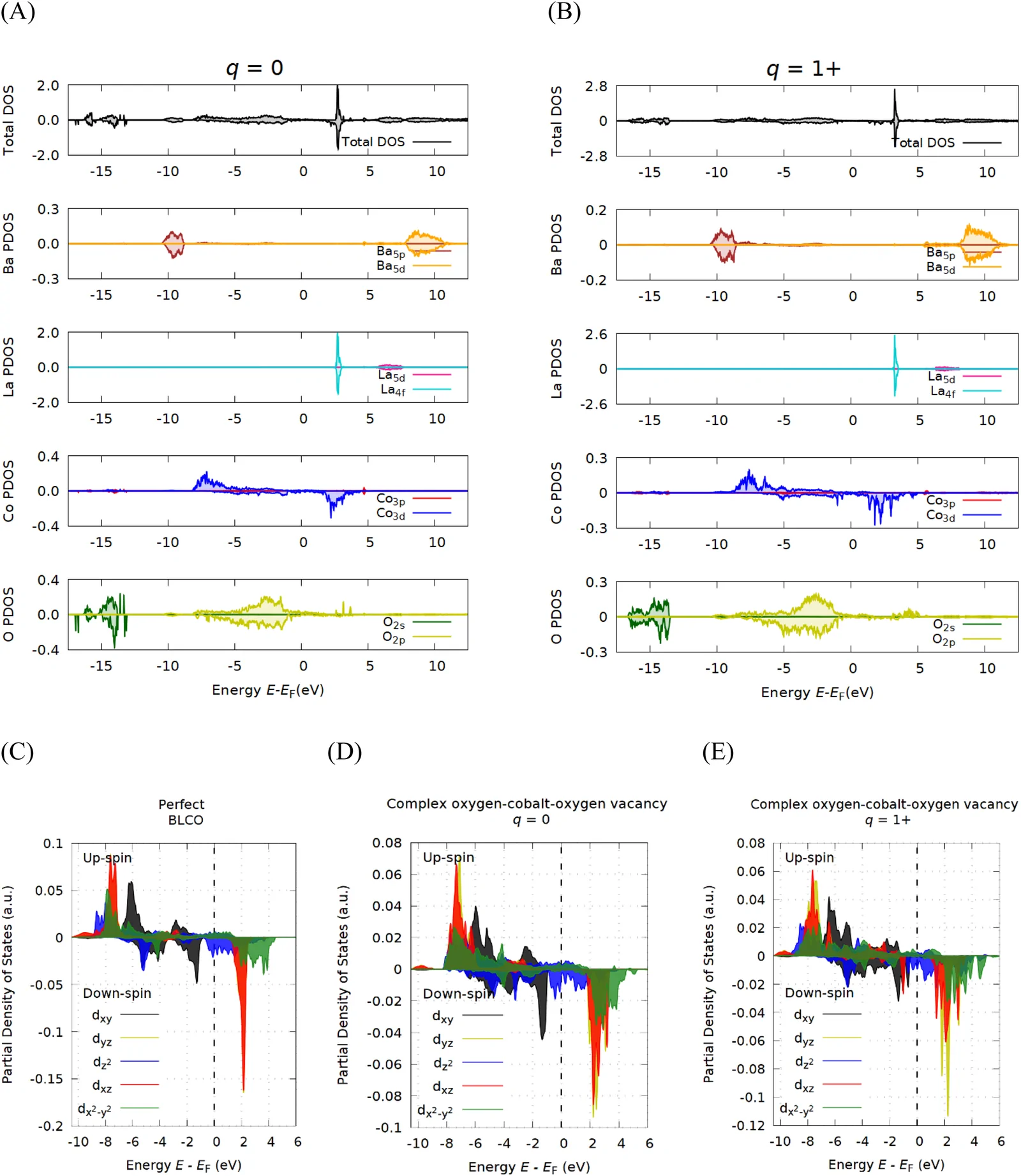
Influence of the complex oxygen–cobalt–oxygen vacancy on the electronic structure of the BLCO. The presentation method is the same as in Fig. 9.

The presence of complex oxygen–cobalt–oxygen vacancy also affects the DOS (see Fig. 13 and Table S2). In particular, for *q* = 0 the Co 3s semi-core state has four and six peaks in the up- and down-spin channels, respectively, while in the perfect BLCO there was a single peak. This splitting is still present after charging the defect to *q* = 1+. The splitting is also noted for the Co 3p state, where seven and three lower intensity peaks appear in the up- and down-spin channel of *q* = 0. In *q* = 1+, this splitting is even more pronounced, with thirteen (up-spin) and seven (down-spin) peaks being visible.

The complex defect also causes minor shifts in the energy positioning of the La 5s, Ba 5s, O 2s and Ba 5p semi-core states, shifting them towards *E*_F_ in both *q* = 0 and *q* = 1+. In addition, charging the complex defect increases the width of some features corresponding to semi-core states (see Table S2). For *q* = 0, the width of the Co 3d – O 2p band remains similar to its width in the perfect BLCO. However, in *q* = 2+ this width increases visibly by 0.9 eV.

Moreover, the small bandgap around *E*_F_ that was present in the down-spin channel of a system containing the charged *trans*-O vacancy pair with *q* = 4+, is closed in the system containing the complex defect, for both its charge states *q* = 0 and 1+. Well-visible changes also include a shift of the La 4f peak, which moves towards *E*_F_ by −1.9 eV (*q* = 0) and −1.2 eV (*q* = 1+).

The presence of the complex oxygen–cobalt–oxygen vacancy also influences the occupation of the Co 3d states. These changes are mostly visible in the down-spin channel, where for *q* = 1+ the occupation of the t_2g_ – d_*XZ*_ state increases by 0.13, when compared to the perfect BLCO (see Table S3). For *q* = 0 the most visible change is a 0.15 increase in the occupation of the e_g_ – d_Z^2^_ state. Due to an increase in the occupation of the down-spin channel, the effective magnetic moment of the system containing the complex defect is lower than that of the perfect BLCO (see Table 4). It decreases to 2.37 µ_B_ (*q* = 0) and 2.28 µ_B_ (*q* = 1+).

## 5 Summary and discussion

In this work, we investigated the influence of exsolution-related defects on the properties of BLCO. The first step involved the generation of dedicated norm-conserving pseudopotentials for Ba, La, Co and O atoms, using our novel approach. The developed methodology was validated for the perfect BLCO, through the calculation of its cohesive energy and equation of state, and comparing findings with the experiment. Out of two considered methodologies, namely the standard DFT and DFT + *U*, the latter provided a better description for the BLCO and its strongly correlated electronic states. Our calculations confirmed the half-metallicity of BLCO, providing a picture consistent with an earlier study that utilized the all-electron FP-LAPW + lo method.

In the next step we studied the influence of defects, considering the single oxygen vacancy, trans O-vacancy pair, and the complex oxygen–cobalt–oxygen vacancy, all in charged and neutral state. After performing geometry relaxations, we analyzed the obtained equilibrium configurations, quantifying the influence of defects on the structural, electronic and magnetic properties. Our DFT + *U* calculations showed that all three defects studied considerably affect the properties of BLCO.

In particular, we observed that for the neutral defects (*q* = 0) the increase in volume is the lowest for the single oxygen vacancy (+3.44%), moderate for the *trans*-O vacancy pair (+4.07%), and the highest for the complex oxygen–cobalt–oxygen vacancy defect (+7.50%). This shows that in the defective BLCO with the formula Ba_0.5_La_0.5_Co_1−*γ*_O_3−*δ*_, the presence of the neutral defects causes a lattice expansion, which increases with the total non-stoichiometry *δ* + *γ* (see Fig. S1A, data for the neutral defects) contrary to that, charged defects do not cause such monotonic behavior in terms of *δ* + *γ*. The presence of single oxygen vacancy with *q* = 2+ decreases the volume slightly (−0.40%), the *trans*-O vacancy pair with *q* = 4+ decreases the volume considerably (−5.75%), and the complex oxygen–cobalt–oxygen vacancy with *q* = 1+ increases the volume by +2.96%. However, we concluded that in the case of charged defects the observed change in volume is strongly related to the defect charge, being a linearly decreasing function of *q* (see Fig. S1B).

The above observations could be useful for interpreting the volume changes seen in the exsolution experiments. For example, ref. 4 and 5 reported that the annealing of cobalt-based perovskites in dry N_2_ leads to an increase in the lattice volume which seems to be proportional to the total non-stoichiometry. We observed a similar trend for the neutral defects (see Fig. S1A). In addition, ref. 4 reported that the lattice of cobalt-based perovskites tends to contract first, and then expand, when they are annealed in dry air. In our case, a similar trend is observed for the charged defects (see also Fig. S1A).

Our calculations revealed that the charge redistribution that occurs after defect introduction has in the BLCO a rather complex character. For all studied defects, we observed a mixed picture, where both localization and delocalization occurred at the same time, but with different intensities. We attribute the simultaneous occurrence of these effects to two competing underlying mechanisms. The observed delocalization can be associated with the half-metallicity of the BLCO, and the tendency to populate its partially-filled down-spin conduction band. The noted localization is a consequence of the tendency of the Co atoms to undergo reduction.

In this regard, our theoretical findings contradict the defect chemistry, which predicts a strong Co reduction (see eqn (2), (4) and (6)). In our DFT + *U* calculations, we observed only a mild reduction of Co atoms, which are nearest to the defect. To support this statement, we note that the charge of the most reduced Co atom changed by approximately −0.27. Such a change was observed for two defects, namely the *trans*-O vacancy and complex oxygen–cobalt–oxygen, both in the neutral state (*q* = 0).

Our observation regarding the delocalized nature of charge redistribution is in agreement with the results presented in ref. 36 and 44. These studies demonstrated that in cobaltite perovskites the introduction of an oxygen vacancy does not lead to the formation of a localized electronic state, but instead results in a pronounced charge delocalization that extends to further coordination shells.

In addition, our DFT + *U* results show that in BLCO the charge redistribution also involves changes in spins of many Co and O atoms. This causes the introduction of defects to considerably alter the magnetic properties of the BLCO. We found that the effective magnetic moment of BLCO, which in the perfect system is 3.0 µ_B_, may decrease to 2.28 µ_B_ or increase to 3.36 µ_B_. Such changes are caused by the complex oxygen–cobalt–oxygen vacancy with *q* = 1+ and *trans*-O vacancy pair with *q* = 0, respectively.

The observed changes in the magnetic properties are a consequence of both identified charge redistribution mechanisms, which lead to changes in the spins of many Co and O atoms, even those that are far from the defect site. We found that in the studied systems, the number of atoms that actively participate in the observed spin redistribution is high, reaching up to 18 atoms. This complex character prevents providing a simple description of the observed effect, and identifying its underlying physical mechanisms. However, we note that the observed changes in magnetic properties cannot be attributed solely to the changes in the spins of Co atoms and their magnetic interactions, as the observed spin redistribution involves many O atoms.

We also found that the considered defects affect the electronic structure of the BLCO. Most visible changes include the splitting of the Co 3s and 3p peaks in the DOS. This phenomenon is absent in the perfect BLCO but appears in defective systems, being more intense for larger defects, *i.e. trans*-O vacancy pair and complex oxygen–cobalt–oxygen vacancy. In addition, the presence of a single oxygen vacancy in its both charge states causes a visible, 0.3–0.5 eV, splitting of the La 4f peaks. This splitting is not present in the perfect BLCO and does not occur for other considered defects. Therefore, we suppose that this splitting phenomenon can be used in a spectroscopic identification of single oxygen vacancies in BLCO. We also found that the charged *trans*-O vacancy pair changes the conductive properties of the BLCO, by introducing a small 0.6 eV bandgap in the down-spin channel.

## Author statement

Francis Oseko: conceptualization, investigation, data curation, writing original draft, writing-review & editing. Aleksandra Mielewczyk-Gryń: conceptualization, supervision, funding acquisition, writing-review & editing. Szymon Winczewski: conceptualization, investigation, supervision, funding acquisition, writing original draft, writing-review & editing.

## Conflicts of interest

The authors declare that they have no known competing financial interests or personal relationships that could have appeared to influence the work reported in this paper.

## Supplementary Material

RA-OLF-D6RA05408K-s001

RA-OLF-D6RA05408K-s002

## Data Availability

The authors confirm that the data supporting the findings of this study are available within the article and its supplementary information (SI). Supplementary information is available. See DOI: https://doi.org/10.1039/d6ra05408k.
