# Supplementary material for: Vacancy-induced modifications to the electronic structure of barium lanthanum cobaltite: density functional theory calculations
Source: RSC Adv. 2026 Sep 14;16(44):54883–903. doi: 10.1039/d6ra05408k (PMC13573168; doi:10.1039/d6ra05408k)
Supplement: Supplementary file 1 [file RA-016-D6RA05408K-s001.pdf]

Supplementary Information (SI) for RSC Advances.
This journal is © The Royal Society of Chemistry 2026

Vacancy-induced modifications to the electronic structure of barium lanthanum cobaltite: density functional theory calculations

Francis Oseko [a*], Aleksandra Mielewczyk-Gryń [a], Szymon Winczewski [a]

[a] Institute of Nanotechnology and Materials Engineering, Gdańsk University of Technology, Gabriela Narutowicza 11/12, 80-233 Gdańsk, Poland

Corresponding Authors [a*]:

Email address: *francis.oseko@pg.edu.pl (Francis Oseko)*

Supplementary Information

1 | Pseudopotentials used

In the attached ZIP archive, we provide the dedicated pseudopotentials which we generated and used in this work, in all carried out DFT and DFT+U calculations. We provide pseudopotentials for the Ba, La, Co and O atoms. Each pseudopotential is provided in two forms: as a RECPOT file and as a PARAM file. The latter can be used to generate the pseudopotential in different (than RECPOT) formats, and this can be done with the OPIUM code[1]. We note that we used OPIUM version 3.8.

2 | Additional results

In this section we provide additional results, which support finding from the main text. In Table S1 we summarize the results of the carried out population analysis, which was performed with the Mulliken method. For each defective system we present various statistical measures describing the changes seen in atomic charges q and spins s, as well as changes in bond lengths d and their occupations f.

In our analysis, we first performed population analysis for the perfect BLCO and defective systems, and then calculated the changes for individual atoms and bonds. For example, in analysis of atomic charges we used the following formula: $\Delta q(i) = q_{\text{defective}}(i) - q_{\text{perfect}}(i)$. Here, $q_{\text{defective}}(i)$ and $q_{\text{perfect}}(i)$ represent the Mulliken charge of i-th atom in the defective and perfect system, respectively. This difference was calculated for each Co and O atom in the system. In Table S1 we report on the minimum (denoted as min), maximum (max), mean (mean), and mean absolute value (mean abs.) of $\Delta q(i)$ and $\Delta s(i)$.

We also provide the number of atoms which display change greater than a specified threshold. The taken thresholds (0.06 for charge and 0.12 for spin) were determined by calculating mean absolute value, averaging over all atoms and all defective systems. Based on these thresholds we determined the numbers of atoms, which display significant change in charge (denoted with N_q), spin (N_s), or both (N_{qs}).

Similar analysis was performed for the Co-O bonds, considering (instead of charge and spin) bond lengths d and their occupations f obtained from the population analysis. The numbers of bonds displaying significant change in length, occupation and both are denoted with N_d, N_f, and N_{df}, respectively. In identifying considerably affected bonds we used the following thresholds: 0.038 Å for bond lengths and 0.033 for occupations. These thresholds were also determined by calculating mean absolute value, with averaging performed over all Co-O bonds and all defective systems.

In the attached ZIP archive, we also provide XYZ files for all studied systems: the perfect BLCO and all defective systems. The provided structures correspond to equilibrium structures obtained from the energy minimization. For each atom in the system we provide its name and three Cartesian coordinates

(expressed in Å). The naming of atoms is the same as in the main text. In the extra four columns, we also provide the calculated atomic charge $q(i)$ and spin $s(i)$, as well as the $\Delta q(i)$ and $\Delta s(i)$ changes defined above. We note that the information about the vectors spanning the supercell is provided in Table 4 in the main text.

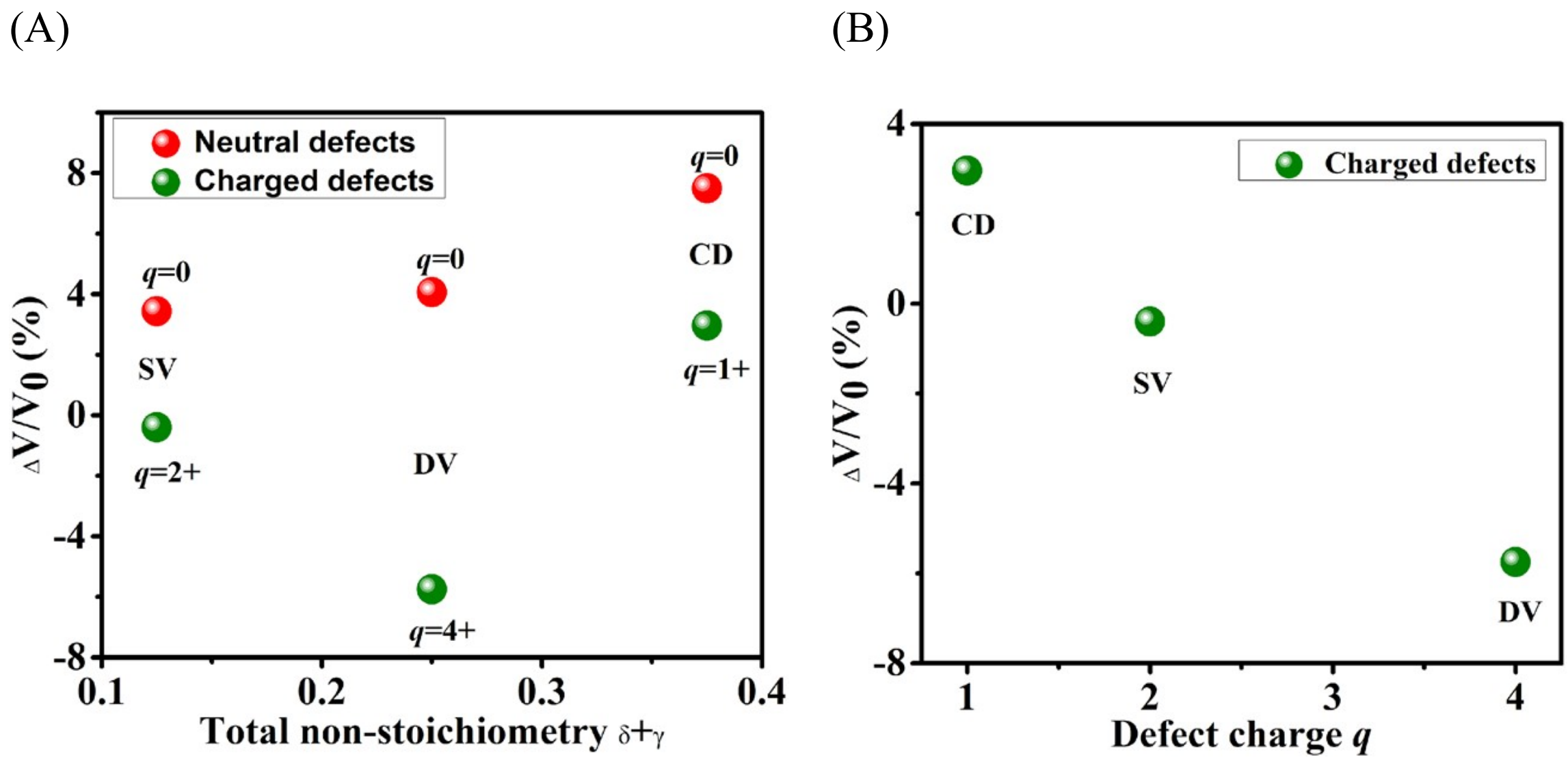

Figure S1. Influence of defects on the volume of BLCO. Both panels show the relative change in volume ($\Delta V/V_0$), presenting it as a function of the total non-stoichiometry (panel A) and defect charge q (panel B). Labels given within plots describe defects, where SV stands for the single oxygen vacancy, DV denotes the trans-O vacancy pair, and CD represents the complex oxygen-cobalt-oxygen vacancy. The change in volume of defective systems ΔV is measured relative to the perfect BLCO, which has volume V_0.

Table S1. Summary of the performed Mulliken population analysis. In the subsequent columns we present results obtained for various studied defects.

Atom/bond type	Metric	Single oxygen vacancy				Trans-O vacancy pair				Complex oxygen-cobalt-oxygen vacancy			
		q = 2+		q = 0		q = 4+		q = 0		q = 1+		q = 0	
		Δq	Δs	Δq	Δs	Δq	Δs	Δq	Δs	Δq	Δs	Δq	Δs
**Co**	min	-0.141	-0.643	-0.168	-0.248	-0.099	-0.513	-0.269	-0.168	-0.148	-0.514	-0.273	-0.393
	max	0.116	0.125	0.095	0.209	0.153	0.154	0.129	0.297	0.123	0.268	0.087	0.112
	mean	0.007	-0.228	0.002	-0.004	0.077	-0.045	0.000	0.102	0.029	0.001	-0.003	-0.054
	mean abs.	0.091	0.295	0.086	0.128	0.126	0.212	0.135	0.186	0.087	0.289	0.077	0.117
	N_q and N_s	6	5	4	4	8	6	6	8	4	6	2	2
	N_{qs}	8		4		8		8		6		3	
		Δq	Δs	Δq	Δs	Δq	Δs	Δq	Δs	Δq	Δs	Δq	Δs
**O**	min	-0.011	-0.711	-0.062	-0.675	0.011	-0.066	-0.089	0.014	-0.055	-1.084	-0.084	-0.863
	max	0.242	0.051	0.177	0.138	0.068	0.048	-0.026	0.188	0.277	0.264	0.273	0.091
	mean	0.038	-0.125	-0.026	-0.012	0.043	0.007	-0.055	0.096	0.012	-0.121	-0.013	-0.088
	mean abs.	0.040	0.145	0.041	0.073	0.043	0.031	0.055	0.096	0.086	0.255	0.062	0.153
	N_q and N_s	4	8	5	3	10	0	8	5	4	10	4	5
	N_{qs}	9		7		10		13		10		7	
		Δd	Δf	Δd	Δf	Δd	Δf	Δd	Δf	Δd	Δf	Δd	Δf
**Co-O**	min	-0.038	-0.120	0.011	-0.140	-0.095	-0.010	0.007	-0.050	-0.082	-0.180	-0.009	-0.160
	max	-0.005	0.080	0.028	0.040	0.0478	0.120	0.053	0.070	0.163	0.100	0.106	0.070
	mean	-0.027	0.014	0.022	-0.012	-0.037	0.036	0.028	-0.007	0.032	-0.020	0.052	-0.023
	mean abs.	0.027	0.028	0.022	0.025	0.049	0.038	0.028	0.024	0.065	0.061	0.057	0.042
	N_d and N_f	30	10	0	10	28	20	16	8	26	28	28	17
	N_{df}	34		10		28		20		34		30	

Table S2. Positioning of features visible in the DOS of the perfect and defective BLCO. A single value indicates the presence of a single narrow peak. More than one value points to multiple narrow peaks. If range is given, then the corresponding DOS feature extends between the given values. All reported positions are in eV and are measured with respect to the Fermi energy.

Feature		Perfect BLCO		Single oxygen vacancy		Trans oxygen vacancy pair		Complex oxygen-Cobalt-oxygen vacancy	
		standard DFT	DFT+U	*q* = 0	*q* = 2+	*q* = 0	*q* = 4+	*q* = 0	*q* = 1+
**Co 3s**	up-spin	-98.2	-97.3	-98.9, -98.2, -97.2	-98.8, -98.5, -97.4	-99.0, -98.2, -97.3	-98.9, -96.4, -96.1	-98.2, -98.0, -97.8, -96.8	-99.2, -98.7, -98.4, -98.2, -97.8, -96.2
	dn-spin	-94.8	-94.6	-94.8, -94.5, -93.9	-94.9, -94.7, -94.3	-94.8, -94.5, -93.6	-94.9, -93.3	-94.5, -94.4, -94.3, -94.2, -93.8, -93.5	-95.0, -94.8, -94.6, -94.4, -94.3, -93.8
**Co 3p**	up-spin	-62.3, -61.1	-60.5	-63.1, -62.2 to -62, -61.6, -61, -60.8, -59.7	-62.9, -62.6, -61.6 to -61.2, -60.8, -59.9	-63.3, -62.2, -61.7 to -61.5, -61.2, -60.8, -59.7	-63.1, -61.7, -60.6, -60, -59.9, -58.3, -58.2	-62.3, -62.2 to -61.8, -61.4 to -60.8, -60.5, -60.2, -60.0, -59.0	-62.8, -62.6, -62.3, -62.2, -61.7, -61.6, -61.4, -61.1, -60.4, -60.2, -60, -58.9, -58.3
	dn-spin	-58.2, -57.9	-58.0	-58.5, -57.9, -57.7 to -57.5, -57.2, -57.0	-58.4, -58.2, -57.9 to -57.7, -57.5, -57.2	-58.5, -58.0 to -57.8, -57.6 to -57.4, -57.1, -56.6	-58.5, -58.0, -57.8, -57.1, -56.7, -56.3	-57.9 to -57.1, -56.8, -56.5	-58.7, -58.3, -58.1 to -57.6, -57.4 to -57.2, -56.8, -56.6, -56.2
**La 5s**		-31.4 to -31.2	-31.6 to -31.5	-31.7 to -31.4	-30.9 to -30.6	-31.6 to -31.2	-30	-31.8	-31.3
**Ba 5s**		-25.0 to -24.6	-24.0 to -23.8	-24 to -23.5	-23.4 to -22.8	-23.9 to -23.4	-22.4	-23.9	-23.7
**O 2s**		-19.0 to -14.8	-17.5 to -14.2	-17.2 to -14.0	-17.4 to -13.7	-16.8 to -13.5	-16.2 to -12.7	-17.1 to -13.8	-17.4 to -13.5
**Ba 5p**		-10.8 to -10.0	-10.5 to -9.0	-10.2 to -8.9	-10.1 to -7.2	-10.4 to -8.4	-10.0 to -7.5	-10.3 to -9.1	-10.4 to -8.6
**Co 3d**		-7.4 to +4.2	-9.0 to +4.6	-8.8 to +4.8	-10.0 to +4.9	-9.4 to +4.7	-10.0 to +5.0	-9.0 to +4.8	-8.9 to +5.0
**O 2p**		-7.5 to +4.2	-10.0 to +4.6	-10 to +3.8	-10.0 to +4.8	-10.0 to +4.7	-9.8 to +4.9	-10.0 to +4.6	-10.2 to + 5.3
**La 4f**		+4.3	+4.6	+2.7 to +3.4	+3.6 to +4.4	+2.8 to +3.5	+4.6	+2.7	+3.4
**La 5d**		+5.1 to +6.7	+6.0 to +7.8	+6.2 to +7.6	+6.6 to +8.5	+6.4 to +7.6	+7.5 to +9.2	+5.8 to +7.5	+6.4 to 8.0
**Ba 5d**		+7.5 to +9.6	+8.5 to +10.5	+7.9 to +10.8	+8.8 to +11.9	+8.0 to +11.0	+9.5 to +12.5	+8.0 to +10.6	+8.2 to +11.3

Table S3. Calculated occupations of the Co 3d - t_{2g} states (d_{XY}, d_{XZ} and d_{YZ}) and e_g states ($d_{X^2-Y^2}$ and d_{Z^2}) in the perfect and defective BLCO. The presented values were obtained by averaging over all Co atoms.

		Perfect BLCO		Defective BLCO					
		Standard DFT	DFT+U	Single oxygen vacancy		Trans oxygen vacancy pair		Complex oxygen-cobalt-oxygen vacancy	
				$q = 0$	$q = 2+$	$q = 0$	$q = 4+$	$q = 0$	$q = 1+$
**Up-spin**	d_{XY}	0.9959	1.0002	0.9996	0.9997	0.9997	0.9980	0.9996	0.9996
	d_{YZ}	0.9959	1.0010	1.0009	1.0010	1.0010	1.0003	1.0008	1.0008
	d_{XZ}	0.9963	1.0010	1.0006	1.0008	1.0007	1.0007	1.0006	1.0005
	d_{Z^2}	0.7899	0.9317	0.9264	0.9138	0.9415	0.8661	0.9302	0.9393
	$d_{X^2-Y^2}$	0.7874	0.9185	0.9316	0.9031	0.9445	0.8896	0.9252	0.9273
**Down-spin**	d_{XY}	0.6396	0.9981	0.9970	0.9968	0.9970	0.9971	0.9937	0.9893
	d_{YZ}	0.6449	0.0698	0.0612	0.0769	0.0614	0.3231	0.0502	0.0570
	d_{XZ}	0.6342	0.0698	0.0543	0.0705	0.0614	0.0767	0.0525	0.1996
	d_{Z^2}	0.3414	0.5349	0.6031	0.5742	0.5466	0.4074	0.6809	0.4921
	$d_{X^2-Y^2}$	0.3368	0.2171	0.2358	0.2570	0.2493	0.2873	0.2301	0.2413

Table S4: A sensitivity test of the U parameters (U558) chosen. Structural and mechanical properties of the perfect BLCO as predicted for different U variations. The symbols represent: the bulk modulus (B) and its pressure derivative (B'), the total energy (E_0) and volume (V_0) at the equilibrium. We note that the experimental lattice parameter (a_0) corresponds to the room temperature and oxygen non-stoichiometry ($\delta > 0$).

	U parameters (eV)			***B*** **(GPa)**	$\boldsymbol{B'}$	$\boldsymbol{E_0}$ **(eV)**	$\boldsymbol{V_0}$ **(Å^3)**	$\boldsymbol{a_0}$ **(Å)**	$\boldsymbol{E_{coh}}$ ***(eV)***	$\mu_{Co}(\mu_B)$	μ_{eff} (μ_B)	**Up-channel**
	**La**	**Co**	**O**									**band gap**
**Standard DFT**	**-**	**-**	**-**	**150.87**	**3.79**	**-56200.2**	**476.527**	**3.905**	**-26.6359**	**2.16**	**2.67**	0.83
U458	4	5	8	148.96	3.21	-56116.7	496.520	3.960	-23.5375	2.93	3.00	2.07
U548	5	4	8	153.77	2.65	-56115.9	494.746	3.955	-23.4089	2.93	3.10	2.03
U556	5	5	6	141.07	4.60	-56122.6	500.213	3.969	-22.0214	2.88	3.00	1.84
U557	5	5	7	143.20	4.61	-56117.6	498.299	3.964	-22.5232	2.91	3.00	1.93
**U558**	**5**	**5**	**8**	**140.03**	**4.79**	**-56112.7**	**496.678**	**3.951**	**-22.9516**	**2.96**	**3.00**	1.88
U559	5	5	9	150.34	3.54	-56108.1	495.465	3.956	-23.5891	2.97	3.03	2.13
U5510	5	5	10	149.06	3.96	-56103.5	492.709	3.949	-24.1407	3.21	3.48	2.71
U568	5	6	8	144.90	4.55	-56109.9	498.869	3.966	-22.7129	2.97	3.00	2.04
U658	6	5	8	146.35	3.40	-56108.6	500.749	3.970	-22.5263	2.93	3.00	1.95
**Experiment**[1]				**153.00**	**-**	**-**	**468.916**	**3.885**	**-22.1931**			

References

1. Opium - pseudopotential generation project. http://opium.sourceforge.net/, Accessed 04-12-2025.
